# *Echinacea purpurea* Fractions Represent Promising Plant-Based Anti-Inflammatory Formulations

**DOI:** 10.3390/antiox12020425

**Published:** 2023-02-09

**Authors:** Sara F. Vieira, Samuel M. Gonçalves, Virgínia M. F. Gonçalves, Carmen P. Llaguno, Felipe Macías, Maria Elizabeth Tiritan, Cristina Cunha, Agostinho Carvalho, Rui L. Reis, Helena Ferreira, Nuno M. Neves

**Affiliations:** 1 3B’s Research Group, I3BS—Research Institute on Biomaterials, Biodegradables and Biomimetics, University of Minho, Headquarters of the European Institute of Excellence on Tissue Engineering and Regenerative Medicine, AvePark, Parque de Ciência e Tecnologia, Zona Industrial da Gandra, Barco, 4805-017 Guimarães, Portugal; 2 ICVS/3B’s–PT Government Associate Laboratory, Braga, Guimarães, Portugal; 3 Life and Health Sciences Research Institute (ICVS), School of Medicine, University of Minho, Campus de Gualtar, 4710-057 Braga, Portugal; 4 TOXRUN—Toxicology Research Unit, University Institute of Health Sciences, CESPU, CRL, 4585-116 Gandra, Portugal; 5 UNIPRO—Oral Pathology and Rehabilitation Research Unit, University Institute of Health Sciences (IUCS), CESPU, CRL, 4585-116 Gandra, Portugal; 6 Departamento de Edafoloxía e Química Agrícola, Facultade de Bioloxía, Universidade de Santiago de Compostela, 15782 Santiago de Compostela, Spain; 7 Interdisciplinary Centre of Marine and Environmental Research (CIIMAR), University of Porto, Terminal de Cruzeiros do Porto de Leixões, Avenida General Norton de Matos, S/N, 4450-208 Matosinhos, Portugal; 8 Laboratório de Química Orgânica e Farmacêutica, Departamento de Ciências Químicas, Faculdade de Farmácia da Universidade do Porto, Rua Jorge de Viterbo Ferreira 228, 4050-313 Porto, Portugal

**Keywords:** *Echinacea purpurea* extracts, fractions, phenols/carboxylic acids, alkylamides, inflammation, human primary macrophages

## Abstract

*Echinacea purpurea* is traditionally used in the treatment of inflammatory diseases. Therefore, we investigated the anti-inflammatory capacity of *E. purpurea* dichloromethanolic (DE) and ethanolic extracts obtained from flowers and roots (R). To identify the class of compounds responsible for the strongest bioactivity, the extracts were fractionated into phenol/carboxylic acid (F1) and alkylamide fraction (F2). The chemical fingerprint of bioactive compounds in the fractions was evaluated by LC-HRMS. *E. purpurea* extracts and fractions significantly reduced pro-inflammatory cytokines (interleukin 6 and/or tumor necrosis factor) and reactive oxygen and nitrogen species (ROS/RNS) production by lipopolysaccharide-stimulated primary human monocyte-derived macrophages. Dichloromethanolic extract obtained from roots (DE-R) demonstrated the strongest anti-inflammatory activity. Moreover, fractions exhibited greater anti-inflammatory activity than whole extract. Indeed, alkylamides must be the main compounds responsible for the anti-inflammatory activity of extracts; thus, the fractions presenting high content of these compounds presented greater bioactivity. It was demonstrated that alkylamides exert their anti-inflammatory activity through the downregulation of the phosphorylation of p38, ERK 1/2, STAT 3, and/or NF-κB signaling pathways, and/or downregulation of cyclooxygenase 2 expression. *E. purpurea* extracts and fractions, mainly DE-R-F2, are promising and powerful plant-based anti-inflammatory formulations that can be further used as a basis for the treatment of inflammatory diseases.

## 1. Introduction

Inflammation is crucial for the survival and maintenance of human health [1]. The inflammatory response is coordinated by the activation of several inflammatory signaling pathways in tissue-resident and recruited immune cells [2]. The main inflammatory signaling pathways associated with the initiation and progression of inflammation are nuclear factor-kappa B (NF-κB) [3], mitogen-activated protein kinase (MAPK) family (extracellular signal-regulated kinase (ERK), C-Jun N-terminal kinase/stress-activated protein kinase (JNK/SAPK), and p38 kinase) [4], cyclooxygenase (COX)-2 expression [5], and Janus kinase/signal transducers and activators of transcription (JAK/STAT) [6].

The dysregulation of the magnitude or duration of the inflammatory response can lead to chronic inflammation, which is characterized by the continuous infiltration of immune cells into the injured tissue [7]. Particularly, macrophages are key mediators of inflammation, orchestrating the immune response. Those cells are responsible for engulfing damaged cells and invading pathogens and present antigens to the adaptive immune system [8]. Once activated, macrophages release high levels of pro-inflammatory mediators, including reactive oxygen and nitrogen species (ROS/RNS) and cytokines (e.g., interleukin (IL)-6 and tumor necrosis factor (TNF)-α) [9,10,11]. These molecules allow for the communication between immune cells, regulating the intensity and duration of the inflammatory response. Hence, their suppression can be a valuable hallmark in the therapy of chronic inflammation where the immune system is overactivated.

The most severe and deleterious outcome of chronic inflammation is the continuous damage and destruction of tissues and organs, which leads to an increased risk of several pathologies (e.g., autoimmune disorders) [12,13]. The current treatment for chronic inflammation-associated diseases varies with their severity, but often it focuses on reducing the overactivity of the immune system. Available anti-inflammatory drugs include nonsteroidal anti-inflammatory drugs (NSAIDs, e.g., celecoxib), corticosteroids (e.g., dexamethasone), conventional disease-modifying anti-rheumatic drugs (cDMARDs, e.g., methotrexate), and biological (b) DMARDs (e.g., anti-IL-6 and anti-TNF-α) [14,15,16]. However, the prolonged administration of these drugs is frequently associated with several serious side effects. Those include disturbances in the gastrointestinal tract and an increased incidence of opportunistic infections and cancer [17,18]. Therefore, there is an urgent need to discover effective and safe anti-inflammatory drugs.

Plants have been an excellent resource of unique compounds with an important role in the development of many therapeutics [19]. Particularly, *Echinacea purpurea* formulations, recognized as safe by the World Health Organization, have been traditionally used as a potent immunomodulatory medicines [20]. Indeed, *E. purpura* extracts are employed to reduce oxidative stress and inflammation, as well as to prevent cold and flu. Moreover, the ability of *E. purpurea* to interact with immune cells is leading to new insights about its anticancer properties [21]. Other biological properties, such as antifungal, antiviral, and antibacterial activities, have also been reported [22]. Particularly, the antioxidant and anti-inflammatory activities of *E. purpurea* have been associated with its ability to reduce the production of ROS/RNS and pro-inflammatory mediators [23], decrease the infiltration of inflammatory cells [24], and block the receptors of the immune cell [25]. The anti-inflammatory properties have been attributed to alkylamides [26,27,28,29,30,31], polysaccharides [32,33,34,35,36], and caffeic acid derivatives [37,38]. More recently, sesquiterpenes have also been proposed as bioactive principles of *E. purpurea* [39]. However, other studies suggested that the anti-inflammatory activity arises from the synergy between the different bioactive classes of compounds present in the *E. purpurea* extracts [40]. Additionally, the mechanism through which *E. purpurea* extracts exert anti-inflammatory activity is still unclear. Although few studies report the cellular mechanism of *E. purpurea* extracts, they are mainly developed in mouse-derived immune cells [32,39,40]. To the best of our knowledge, only two studies evaluated the mechanism of action of *E. purpurea* extracts using human-derived immune cells. Fast et al. prepared an aqueous extract that reduced the TNF-α production via the inhibition of Toll-like receptor (TLR) 1/2 in Pam3Csk4-stimulated human macrophages [33]. Chicca et al. reported that the standardized commercial tincture Echinaforce decreased the TNF-α production in part via cannabinoid type 2 (CB2) receptor signaling in lipopolysaccharide (LPS)-stimulated human peripheral blood mononuclear cells (PBMCs) [41]. Thus, the understanding of how a particular bioactive class of compounds present in *E. purpurea* extracts produces its effects in human-derived primary cells, which mimic the human cell environment, is urgently needed. Bioactivity-guided fractionation assays will help in the identification of substances responsible for the biological activity.

In a previous study, we demonstrated the potential of several *E. purpurea* extracts to reduce cytokine production and ROS/RNS levels in an LPS-stimulated macrophage cell line [42]. In this work, we aim to corroborate their anti-inflammatory effects with human primary monocyte-derived macrophages (hMDMs), investigate the bioactive principles, and explore the therapeutic targets. The three most promising extracts in the previous study—dichloromethanolic extracts obtained from roots (DE-R), dichloromethanolic extracts obtained from flowers (DE-F), and ethanolic extracts obtained from flowers (EE-F)—were selected for this work [42]. The *E. purpurea* extracts prepared using a green and innovative extraction technique, the Accelerated Solvent Extractor (ASE), were fractionated by semi-preparative high-performance liquid chromatography (HPLC) into a phenol/carboxylic acid rich fraction (F1) and an alkylamide rich fraction (F2) to identify the class of compounds responsible for the strongest bioactivity. Moreover, the chemical fingerprint of the bioactive compounds in the fractions was also evaluated by liquid chromatography–high-resolution mass spectrometry (LC-HRMS). The reduction in the production of pro-inflammatory cytokines (IL-6 and TNF-α), the decrease in intracellular ROS/RNS generation, and the downregulation of inflammatory signaling pathways (NF-κB, ERK1/2, p38, JNK/SAPK, STAT3, COX-2) were investigated in LPS-stimulated primary human monocyte-derived macrophages (hMDMs). LPS is an exogenous stimulus derived from the cell wall of Gram-negative bacteria that promotes the release of pro-inflammatory mediators (e.g., cytokines and ROS/RNS) [43]. After fractionation of *E. purpurea* extracts, it was observed that F2 enhanced the anti-inflammatory activity, suggesting that alkylamides are the bioactive compounds mainly responsible for this bioactivity. Interestingly, the further fractionation of alkylamides fraction demonstrated the existence of a possible synergistic effect between them. To the best of our knowledge, this is the first study demonstrating the anti-inflammatory effects of *E. purpurea*, mainly of dichloromethanolic extracts and their fractions, in LPS-stimulated hMDM, through the suppression of ERK1/2, p38, STAT3, and COX-2 inflammatory signaling pathways.

## 2. Materials and Methods

**Figure 1:**
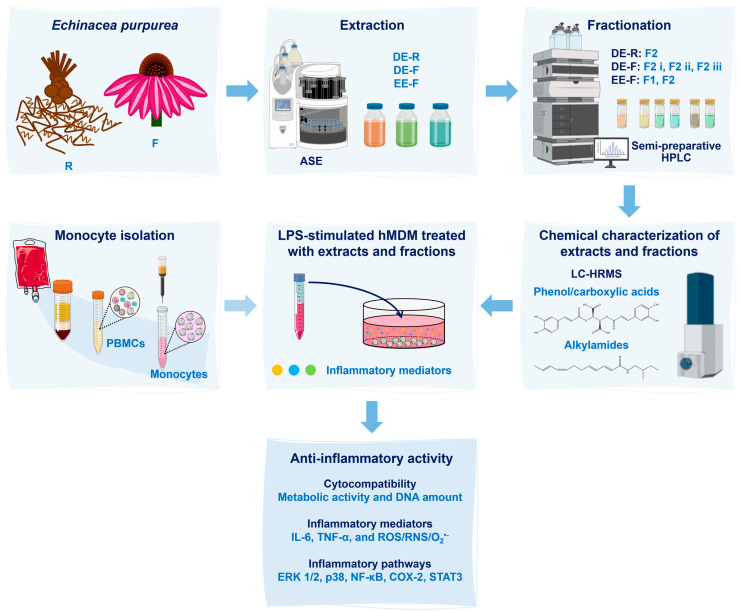
Scheme of the experimental procedure used in this work. *Echinacea purpurea* root (R) and flower (F) extracts were obtained using an Accelerated Solvent Extractor (ASE). Three extracts were prepared: dichloromethanolic extracts obtained from roots (DE-R), dichloromethanolic extracts obtained from flowers (DE-F), and ethanolic extracts obtained from flowers (EE-F). Then, the extracts were fractionated into phenol/carboxylic acid fractions (F1) and alkylamide fractions (F2) by semi-preparative high-performance liquid chromatography (HPLC). Both extracts and fractions were chemically characterized by liquid chromatography–high-resolution mass spectrometry (LC–HRMS). After, the whole extracts and fractions, at different concentrations, were added to lipopolysaccharide (LPS)-stimulated human monocyte-derived macrophages (hMDMs). These cells were isolated from human blood. Their cytocompatibility was evaluated through the metabolic activity and DNA amount determination. Their anti-inflammatory activity was validated by the decrease in interleukin (IL)-6 and tumor necrosis factor (TNF)-α levels in the cell culture medium, as well as by the reduction in the intracellular generation of ROS/RNS/O_2_^•−^. Moreover, inflammatory pathways, including ERK1/2, p38, NF-κB, COX-2, and STAT3, were analyzed to determine their mechanisms of action.

A scheme detailing the sequence of the methodology used in this work is illustrated in Figure 1.

### 2.1. Reagents and Chemicals

*E. purpurea* was purchased from Cantinho das Aromáticas (Vila Nova de Gaia, Portugal) in May 2017. The plants were transferred to soil and grown following a sustainable agriculture procedure (41°37′04.5″ N, 7°16′14.4″ W). After two years of cultivation, the flowers were collected in a full bloom phase (June and July 2019), and the roots, including rhizomes, were harvested in the autumn (October 2019). Flowers and roots were dried in the dark and stored at room temperature (RT) and protected from light and humidity until further use. HPLC-grade dichloromethane, acetonitrile (ACN) and HPLC-grade methanol were obtained from Fisher Scientific, Portugal. Dimethyl sulfoxide (DMSO) was purchased from VWR, Portugal. Roswell Park Memorial Institute (RPMI)-1640 media, 4-(2-hydroxyethyl)-1-piperazineethanesulfonic acid (HEPES) buffer solution 1 M, penicillin–streptomycin (10,000 U/mL), Dulbecco’s phosphate-buffered saline (DPBS), formalin 10% (*v/v*), Quant-iT PicoGreen dsDNA Kit, Pierce Phosphatase Inhibitor Mini Tablets, PageRuler Plus Prestained Protein Ladder (10 to 250 kDa), Bolt Sample Reducing Agent, Bolt LDS Sample Buffer, Bis-Tris Bolt 8%, Bolt MES SDS Running Buffer, and iBlot 2 Transfer Stacks (polyvinylidene fluoride, PVDF) were purchased from Thermo Fisher Scientific, Lisbon, Portugal. OctoMACS separator, human CD14 microbeads, MS columns, and human recombinant granulocyte–macrophage colony-stimulating factor (GM-CSF) were obtained from Miltenyi Biotec, Bergisch Gladbach, Germany. AlamarBlue, Bio-Rad Protein Assay Dye Reagent Concentrate, and Tween 20 were purchased from Bio-Rad, Lisbon, Portugal. Human IL-6 and TNF-α DuoSet Enzyme-linked immunosorbent assay (ELISA) and DuoSet ELISA Ancillary Reagent Kit 2 were purchased from R&D Systems, Minneapolis, MN, USA. Ethanol, formic acid analytical grade, dexamethasone, Histopaque-1077, human serum, lipopolysaccharide (LPS, *Escherichia coli* O26:B6), radioimmunoprecipitation assay (RIPA) buffer, complete mini protease inhibitor cocktail tablets, bovine serum albumin (BSA), Tris-base, and high-purity standards of echinacoside, chicoric acid, caftaric acid, caffeic acid, chlorogenic acid, and cynarin were obtained from Sigma-Aldrich, Lisbon, Portugal. Echinacea isobutylamide standards kit, composed of undeca-2E/Z-ene-8,10-diynoic acid isobutylamide, dodeca-2E-ene-8,10-diynoic acid isobutylamide, and dodeca-2E,4E-dienoic acid isobutylamide, was acquired from ChromaDex, Los Angeles, CA, USA. High-purity standard dodeca-2E,4E,8Z,10E/Z-tetraenoic acid isobutylamide was obtained from Biosynth Carbosynth, Spain. Cellular ROS/Superoxide (O_2_^•−^) detection assay kit and rabbit glyceraldehyde-3-phosphate dehydrogenase (GAPDH) were acquired from Abcam, Boston, MA, USA. IRDye 800CW Goat anti-Rabbit IgG and IRDye 680RD Goat anti-Rabbit IgG secondary antibodies were obtained from LI-COR Biosciences GmbH, Bad Homburg, Germany. Rabbit NF-κB p65, rabbit p44/42 MAPK (ERK 1/2), rabbit p38 MAPK, rabbit SAPK/JNK, rabbit STAT3, rabbit COX-2, rabbit inducible nitric oxide synthase (iNOS), rabbit phospho-NF-κB p65, rabbit phospho-p38 MAPK, rabbit phospho-STAT3, rabbit phospho-SAPK/JNK, and rabbit phospho-p44/42 MAPK (ERK 1/2) were purchased from Cell Signaling Technology, Lisbon, Portugal. Sodium chloride was acquired from PanReac AppliChem, Lisbon, Portugal. Celecoxib was obtained from abcr GmbH, Karlsruhe, Germany. DAPI (4′,6-diamidino-2-phenylindole) was purchased from Biotium, Fremont, CA, USA. Ultra-pure water was obtained from a Milli-Q^®^ Direct Water Purification System (Milli-Q Direct 16, Millipore, Molsheim, France).

### 2.2. Bioactive Compounds Extraction

Dried flowers (F) or roots (R) were ground using an Analytical Sieve Shaker (AS200 Digit, Retsch, Haan, Germany) before extraction. Dichloromethanolic extracts (DE) and ethanolic extracts (EE) were prepared using an Accelerated Solvent Extractor 200 (ASE, Dionex Corp. Vigo, Spain), as previously described by Vieira et al. [42]. Briefly, the mixture of the plant material (2–5 g) with diatomaceous earth was placed and pressed into stainless-steel extraction cells, presenting cellulose filters in the bottom. Two extraction cycles were carried out at constant pressure (1500 psi) for 30 min at the minimal operation temperature of the equipment (40 °C). The extract solutions were collected in vials, and then the organic solvent was evaporated using nitrogen. Once dried, all the extracts were stored at −80 °C until further use.

#### 2.2.1. Fractionation of Extracts

The chromatographic separation of the phenols/carboxylic acids and alkylamides was first optimized with standards by analytical HPLC. A stock solution of 1 mg/mL of all standards was prepared and stored in amber bottles at −80 °C. All standards were prepared in methanol, except the caffeic acid solution, which was prepared in ethanol. A standard mixture was prepared at a final concentration of 100 μg/mL for each. A LaChrom Merck Hitachi system equipped with a D-7000 Interface, an L-7100 Pump, an L-7200 autosampler, an L-7455 diode array detector (DAD), and an HPLC System Manager HSMD-7000 (Merck Hitachi, Tokyo, Japan), version 3.0, was used in the chromatographic analysis. The chromatographic separation was performed on a LiChrocart LiChrosphere 100 RP-18 (250 mm × 4 mm, 5 μm, Merck, Darmstadt, Germany). The gradient elution was optimized following the previous method reported by Pellati et al. [44], the mobile phase being composed of water containing 0.1% formic acid and ACN (Supplementary Table S1). The flow rate was 1 mL/min, and the column was set at RT. The injection volume was 20 μL. The UV spectra were acquired in the range of 190 to 450 nm, and the peak integration was performed at 254 nm for alkylamides and 330 nm for caffeic acid and its derivatives.

The optimized separation method was adapted for the fractionation of *E. purpurea* extracts by semi-preparative HPLC. In order to reduce the time consumed, the previous gradient method was optimized (Table 1). The flow rate was set at 2 mL/min. The injection volume of *E. purpurea* extracts varied between 200 and 400 μL. A Uptisphere WOD homemade semi-preparative column (250 mm × 10 mm, 5 µm, interchrom, Interchim, Montluçon, France) was used.

The dry residues of DE and EE were dissolved in methanol (5 to 25 mg/mL) and centrifuged (10,000× *g*, 5 min; ScanSpeed Mini, Labogene, Lillerød, Denmark) to collect the supernatant. The DE and EE were fractionated into two main fractions: Fraction 1 (F1, 2–11 min) and Fraction 2 (F2, 11–20 min), defined as phenol and carboxylic acid fraction and alkylamide fractions, respectively. Fractions were obtained through the eluent collection. Briefly, the supernatants were injected in the LaChrom Merck Hitachi system equipped with a D-7000 Interface, an L-7100 Pump, an L-7200 autosampler, an L-7455 diode array detector (DAD) and an HPLC System Manager HSMD-7000, version 3.0. The chromatographic separation was performed on an Uptisphere WOD homemade semi-preparative column (250 mm × 10 mm, 5 µm, interchrom, Interchim, France). The mobile phase was composed of (A) water containing 0.1% formic acid and (B) ACN (Table 1). Only F2 (12.2–21.5 min) was recovered from DE-R. DE-F was fractionated into F2-i (12–14.6 min), F2-ii (14.6–16 min), and F2-iii (16–20 min). EE-F was fractionated into F1 (2–11 min) and F2 (11–20 min). The organic solvent was evaporated in a rotavapor (R210 Buchi, Switzerland), and then the fractions were freeze-dried (LyoQuest Plus Eco, Telstar, Terrassa, Spain) to remove the water. The crude fractions were stored at −80 °C until further use.

#### 2.2.2. Characterization of Fractions Composition by LC-HRMS Analysis

The LC-HRMS analysis of the fractions was performed according to the method described by Vieira et al. [42]. Briefly, the LC-HRMS analysis was performed on UltiMate 3000 Dionex ultra-high-performance liquid chromatography (UHPLC, Thermo Scientific, Lisbon, Portugal), coupled to an ultra-high-resolution quadrupole–quadrupole time-of-flight (UHR-QqTOF) mass spectrometer (Impact II, Bruker, Lisbon, Portugal). The chromatographic separation was performed on an Acclaim RSLC 120 C18 analytical column (100 mm × 2.1 mm i.d.; 2.2 µm, Dionex, Lisbon, Portugal). The mobile phase was composed of (A) water containing 0.1% formic acid and (B) ACN containing 0.1% formic acid. The gradient program was as follows: 0 min, 95% A; 10 min, 79% A; 14 min, 73% A; 18.3 min, 42% A; 20 min, 0% A; 24 min, 0% A; 26 min, 96% A. The LC-HRMS acquired data were processed using Bruker Compass DataAnalysis 5.1 software (Bruker, Lisbon, Portugal) to extract the mass spectral features from the sample raw data. Echinacoside, chicoric acid, caftaric acid, caffeic acid, chlorogenic acid, cynarin, undeca-2E/Z-ene-8,10-diynoic acid isobutylamide, dodeca-2E-ene-8,10-diynoic acid isobutylamide, dodeca-2E,4E-dienoic acid isobutylamide, and dodeca-2E,4E,8Z,10E/Z-tetraenoic acid isobutylamide were the standards used to confirm the identity of the compounds present in the fractions. The identification of these compounds in the *E. purpurea* fractions was confirmed by their retention time (*t*_R_, min), the mass-to-charge ratio (*m/z*) of the molecular ion, and MS/MS fragmentation patterns. Supplementary Table S2 presents the characteristics of standards obtained by LC-HRMS. The potential identity of the compound in which *t_R_* and MS data did not match with the available standards were assigned by comparing the MS/MS spectra with the theoretical data MS/MS fragments and data in the literature [44,45,46,47,48,49,50].

### 2.3. Preparation of E. purpurea Extracts and Fractions Solutions

Stock solutions of DE-R, DE-F, and EE-F (30.0 mg/mL) and of F1 and F2 (60.0 mg/mL) were prepared in DMSO. Then, serial dilutions were made with complete RPMI (cRPMI, RPMI-1640 culture medium with 2 mM glutamine supplemented with 10% human serum, 1% penicillin/streptomycin, and 1% HEPES). The final concentrations of the samples in the well were 10, 50, and 100 μg/mL. The fractions were only tested in the highest concentration. The maximum concentration of DMSO in the well (0.33%) did not affect the cell viability.

### 2.4. Human Monocytes

#### 2.4.1. Ethics Statement

The in vitro experiments involved cells isolated from the peripheral blood of healthy volunteers at the Hospital of Braga, Portugal, approved by the Ethics Subcommittee for Life and Health Sciences (SECVS) of the University of Minho, Braga, Portugal (no. 014/015). Experiments were conducted according to the principles expressed in the Declaration of Helsinki, and participants provided written informed consent.

#### 2.4.2. Monocyte Isolation and Differentiation

Monocytes were isolated from the PBMCs of three different donors, as previously described by Gonçalves et al. [51]. Briefly, PMBCs were first subjected to a density gradient centrifugation using a Histopaque-1077 solution. The PBMC ring was carefully collected and washed twice with PBS. Then, the monocytes were isolated from PBMCs using positive magnetic bead separation with CD14 microbeads, according to the manufacturer’s instructions. Isolated monocytes were resuspended in cRPMI. After, monocytes were seeded at a density of 1 × 10^6^ cells/mL in adherent 24-well culture plates for 7 days in the presence of 20 ng/mL of recombinant human GM-CSF, at 37 °C, in a humidified atmosphere of 5% CO_2_. The culture medium was replaced every 3 days, and the acquisition of macrophage morphology was confirmed by visualization under an inverted microscope (Axiovert 40, Zeiss, Göttingen, Germany).

#### 2.4.3. Evaluation of Anti-Inflammatory Activity

The hMDMs were stimulated with 100 ng/mL of LPS in a fresh cRPMI medium. After 2 h, all *E. purpurea* extracts and fractions, at different concentrations (see Section 2.3), were added to the LPS-stimulated hMDMs and incubated for 22 h at 37 °C, in a humidified atmosphere of 5% CO_2_. Afterward, the culture medium was harvested (the triplicates were mixed and homogenized) and stored, aliquoted at −80 °C until cytokine quantification. The cells were washed with warm sterile DPBS, and the metabolic activity and DNA content were determined (see Section 2.4.4). Controls containing the same percentage of DMSO (see Section 2.3) in the maximal concentration of extracts/fractions were also tested. hMDM cultures stimulated or not with LPS were used as negative and positive controls for the production of pro-inflammatory mediators, respectively. Dexamethasone and celecoxib, prepared in ethanol (20 mM) and diluted with cRPMI (10 μM in the well), were used as positive controls of inhibition of the production of the pro-inflammatory mediators.

#### 2.4.4. Metabolic Activity and DNA Quantification

The metabolic activity of hMDM incubated with *E. purpurea* extracts and fractions was determined by the reduction of resazurin (blue) to resorufin (pink) by living macrophages using the alamarBlue assay [43]. These results are expressed in percentages related to the positive control.

The DNA concentration of macrophages was quantified using a fluorometric dsDNA quantification kit, according to the instructions of the manufacturer, as previously described by Vieira et al. [43]. DNA contents are expressed in relative concentrations of the positive control.

#### 2.4.5. Cytokine Measurement

The amounts of IL-6 and TNF-α were assayed using ELISA kits, according to the instructions of the manufacturer. The obtained values were normalized by the respective DNA concentration. The results are expressed in percentage relative to the positive control.

#### 2.4.6. Cellular ROS/RNS/O_2_^•−^ Detection Assay

Oxidative stress in the presence or absence of *E. purpurea* extracts and fractions was investigated using a cellular ROS/O_2_^•−^ detection assay kit, as previously described by Vieira et al. [42]. Briefly, LPS-stimulated hMDMs were treated with *E. purpurea* extracts and fractions, at 100 μg/mL, as mentioned before (see Section 2.4.3). After, the hMDMs were washed and labeled with the oxidative stress detection reagent (green, Ex/Em 490/525 nm) for the determination of total ROS/RNS, and O_2_^•−^ detection reagent (orange, Ex/Em 550/620 nm) for 1 h at 37 °C in the dark. These nonfluorescent detection reagents diffuse into cells, where they can be oxidized by ROS/RNS and O_2_^•−^, converting to fluorescent probes. Then, the cells were fixed with 10% of formalin and the nucleus was labeled with DAPI in a ratio of 1:1000 in DPBS, for 10 min. The fluorescent samples were analyzed using a Fluorescence Inverted Microscope with Incubation (Axio Observer, Zeiss, Germany). The fluorescence intensity values, analyzed using ImageJ software (version 1.52a, Wayne Rasband, National Institutes of Health, Bethesda, MD, USA), were normalized against the number of nuclei. Changes in the fluorescence intensity relative to the positive control were related to an increase or decrease in the generation of intracellular ROS/RNS and/or O_2_^•−^.

#### 2.4.7. Western Blot Analysis

LPS-stimulated hMDMs (5 × 10^5^/well in 24-well plates) were treated with *E. purpurea* extracts and fractions, at 100 μg/mL, as previously described (see Section 2.4.3). After 24 h, the medium was removed, and the cells were washed with ice DPBS. Then, the cells were lysed in RIPA buffer containing a mixture of protease and phosphatase inhibitors at 4 °C for 30 min under shaking. Samples were collected and centrifuged (2000 rpm, 20 min). The supernatant was transferred to a new Eppendorf flask and the protein content was determined using the Bio-Rad Protein Assay, based on the method of Bradford. Bolt sample reducing agent and bolt LDS sample buffer were added to 30 μg of protein. Then, the samples were heated and denatured at 70 °C (20 min) and 95 °C (5 min). The centrifuged samples were loaded and separated on 8% precast polyacrylamide gels set on a Mini Gel Tank (Invitrogen, Thermo Fisher Scientific, Lisbon, Portugal). The proteins were transferred from the gel to a PVDF membrane using the iBlot 2 Gel Transfer Device (Invitrogen, Thermo Fisher Scientific, Lisbon, Portugal).

After blocking for 30 min at RT with 5% BSA in Tris-buffered saline Tween 20 (TBST), the membranes were incubated overnight at 4 °C with the following primary antibodies diluted in blocking solution: rabbit NF-κB p65 (1:1000), rabbit p44/42 MAPK (ERK1/2; 1:1000), rabbit p38 MAPK (1:1000), rabbit SAPK/JNK (1:1000), rabbit STAT3 (1:1000), rabbit COX-2 (1:500), rabbit iNOS (1:500), rabbit phospho-NF-κB p65 (1:1000), rabbit phospho-p38 MAPK (1:1000), rabbit phospho-STAT3 (1:2000), rabbit phospho-SAPK/JNK (1:1000), rabbit phospho-p44/42 MAPK (p-ERK1/2; 1:1000), and rabbit GAPDH (1:10,000). Afterwards, the membranes were washed three times for 5 min with TBST, and then IRDye 800CW Goat anti-Rabbit IgG or IRDye 680RD Goat anti-Rabbit IgG secondary antibodies, both diluted in TBST (1:15,000), were added and the samples were incubated for 1 h at RT in the dark. The Odyssey Fc Imaging System (LI-COR Inc., 2800, Lincoln, NE, USA) was used for image acquisition of the Western blots using near-infrared wavelengths of 700 or 800 nm. The intensity of the bands was quantified with Image Studio software (LI-COR, Inc. software version, Lincoln, NE, USA). The data were normalized to the housekeeping GAPDH.

### 2.5. Statistical Analysis

Results are expressed as mean ± standard deviation (SD) of 3 independent experiments with a minimum of 3 replicates for each condition. Statistical analyses were performed using GraphPad Prism 8.0.1 software (Boston, MA, USA). Two-way analysis of variance (ANOVA) and Dunnett’s multiple comparisons or Sidak’s multiple comparisons test was used for cell assays. Differences between experimental groups were considered significant with a confidence interval of 99% when *p* < 0.01.

## 3. Results

### 3.1. Fractionation of the E. purpurea Extracts

**Figure 2:**
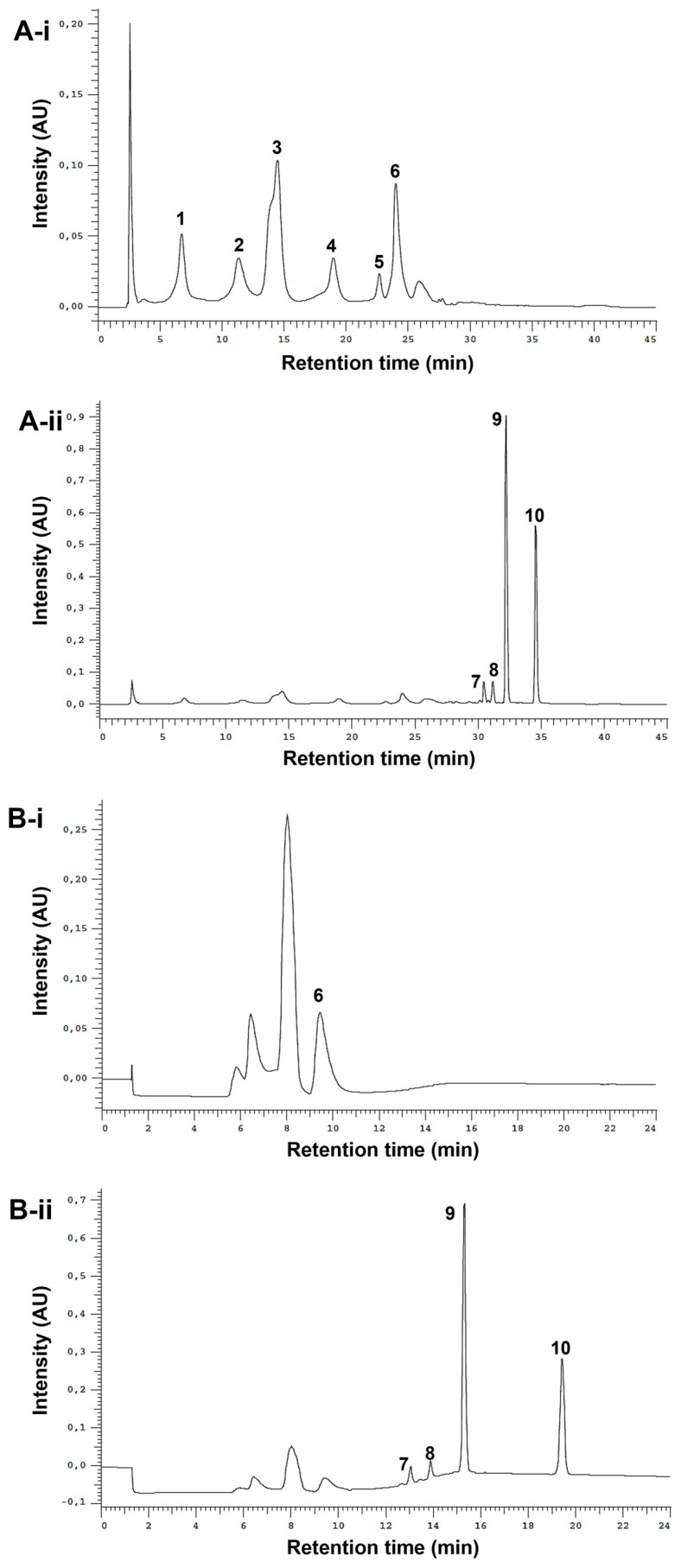
Analytical (**A**) and semi-preparative HPLC chromatograms (**B**) of standard mixture detected at 330 nm (phenols, **i**) and 254 nm (alkylamides, **ii**). Peak numbering on the chromatograms refers to the following compounds: 1—caftaric acid (*t_R_*-A= 6.77 min); 2—chlorogenic acid (*t_R_*-A = 11.36 min); 3—caffeic acid (*t_R_*-A = 14.51 min); 4—cynarin (*t_R_*-A = 18.99 min); 5—echinacoside (*t_R_*-A = 22.72 min); 6—chicoric acid (*t_R_*-A = 24.03 min, *t_R_*-B = 9.47 min); 7—undeca-2E/Z-ene-8,10-diynoic acid isobutylamide (*t_R_*-A = 30.48 min, *t_R_*-B = 13.07 min); 8—dodeca-2E-ene-8,10-diynoic acid isobutylamide (*t_R_*-A = 31.17 min, *t_R_*-B = 13.89 min); 9—dodeca-2E,4E,8Z,10E/Z-tetraenoic acid isobutylamide (*t_R_*-A = 32.24 min, *t_R_*-B = 15.33 min); 10—dodeca-2E,4E-dienoic acid isobutylamide (*t_R_*-A = 34.59 min, *t_R_*-B = 19.44 min).

The optimized analytical method led to the successful separation of the ten studied standards (Figure 2A-i,ii). It was possible to clearly distinguish between phenols and alkylamides. The phenols, due to their high polarity, eluted first under reversed-phase conditions (from ≈ 5 to 28 min), while alkylamides, which are less polar, eluted later (from ≈ 30 to 37 min). To fractionate the extracts, a semi-preparative HPLC method was employed. The analytical method conditions were optimized to reduce the run time and increase the injection volume while maintaining the baseline separation of the two different fractions of interest. In the chromatogram obtained from the standard mixture (Figure 2B-i,ii), it is possible to observe a robust gap between phenol/acids and alkylamide fractions (from 10.6 min to 12.2 min), ensuring the successful separation between these two types of compounds.

**Figure 3:**
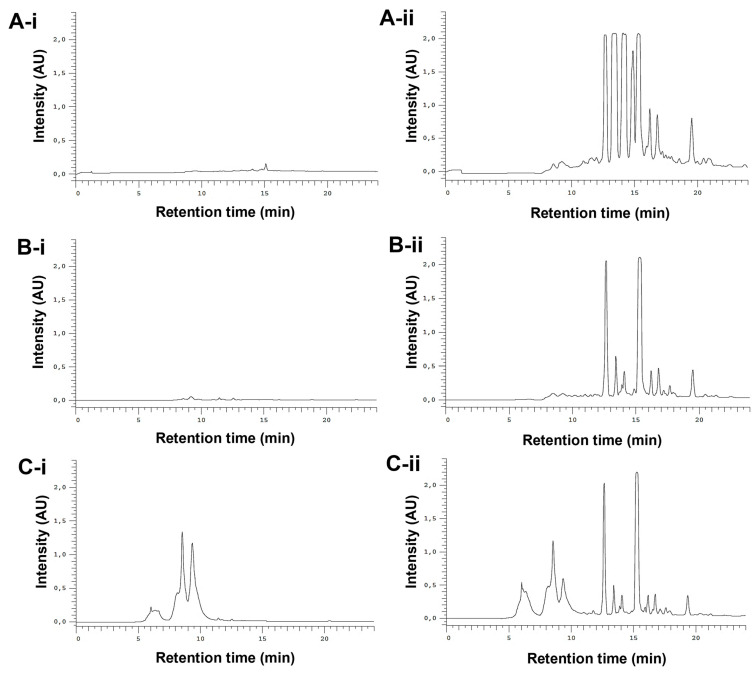
Semi-preparative HPLC chromatograms of DE-R (**A**), DE-F (**B**), and EE-F (**C**) at 19–24.6 µg/mL (100 µL), detected at 330 nm (phenols/carboxylic acids; **A-i,B-i,** and **C-i**) and 254 nm (alkylamides; **A-ii, B-ii,** and **C-ii**).

The whole *E. purpurea* extracts were fractioned into F1 (phenol and carboxylic acid fraction) and F2 (alkylamide fraction). Different chromatogram profiles were observed for DE-R, DE-F, and EE-F (Figure 3). Accordingly, DE-R and DE-F showed higher absorbance values for alkylamides than phenols/carboxylic acids (Figure 3A-i,ii,B-i,ii). Moreover, the first extract seems to be more enriched with alkylamides. EE-F also presented phenols/carboxylic acids and alkylamides in their composition (Figure 3C-i,ii). Based on their chromatographic profiles, the *E. purpurea* extracts were fractionated. As phenols/carboxylic acids were not detected in DE-R, only the F2 fraction was harvested. DE-F presented defined alkylamide peaks, being possible their fractionation into three sections: F2 i showing three clear peaks, F2 ii presenting one perfect peak, and F2 iii displaying three main peaks. Finally, EE-F was fractionated into F1 and F2.

### 3.2. Chemical Composition of the E. purpurea Fractions

The identification of the bioactive compounds present in the *E. purpurea* fractions was performed by LC-HRMS (Table 2). Both product ions and relative intensities for standard fragments perfectly matched those obtained for the compounds in *E. purpurea* fractions. Supplementary Tables S3 and S4 include the retention times (*t*_R_), the precursor ions, and the product ions for phenols/carboxylic acids and alkylamides, respectively. Each extract and fraction exhibited different patterns of phenols/carboxylic acids and alkylamides. The identification of phenols/carboxylic acid compounds and alkylamides in whole DE-R, DE-F, and EE-F was comparable to our previous study [42]. Five phenols/carboxylic acids and twenty-three alkylamides were identified in all *E. purpurea* fractions. As expected, F1 only presented phenols/carboxylic acids, while alkylamides are just observed in F2. EE-F-F1 exhibited five phenols/carboxylic acids in its composition. DE-R-F2 presented the highest number of identified alkylamides (19 compounds), followed by EE-F-F2 (18 compounds), DE-F-F2 i (10 compounds), and DE-F-F2 ii and DE-F-F2 iii (4 compounds).

DE-R-F2 presented the following alkylamides: dodeca-2E,4Z,10E-triene-8-ynoic acid isobutylamide, dodeca-2E,4Z,10Z-triene-8-ynoic acid isobutylamide, dodeca-2E,4E,10Z-triene-8-ynoic acid isobutylamide, dodeca-2Z,4E,10Z-triene-8-ynoic acid isobutylamide, dodeca-2E,4E,10E-triene-8-ynoic acid isobutylamide, undeca-2E,4Z-diene-8,10-diynoic acid isobutylamide, undeca-2Z,4E-diene-8,10-diynoic acid isobutylamide, dodeca-2E,4Z-diene-8,10-diynoic acid isobutylamide, dodeca-2E-ene-8,10-diynoic acid isobutylamide, trideca-2E,7Z-diene-10,12-diynoic acid isobutylamide, dodeca-2,4-diene-8,10-diynoic acid 2-methylbutylamide, dodeca-2Z,4Z,10Z-triene-8-ynoic acid isobutylamide, trideca-2E,7Z-diene-10,12-diynoic acid 2-methylbutylamide, dodeca-2E,4E,8Z,10E/Z-tetraenoic acid isobutylamide, dodeca-2E,4Z,10E-triene-8-ynoic acid 2-methylbutylamide or dodeca-2E-ene-8,10-diynoic acid 2-methylbutylamide, pentadeca-2E,9Z-diene-12,14-diynoic acid isobutylamide, dodeca-2E,4E,8Z-trienoic acid isobutylamide, dodeca-2E,4E,8Z,10E/Z-tetraenoic acid 2-methylbutylamide, and dodeca-2E,4E-dienoic acid isobutylamide.

The fractionation of DE-F originated three different alkylamide fractions, namely (i) DE-F-F2 i composed of dodeca-2E,4Z,10E-triene-8-ynoic acid isobutylamide, dodeca-2E,4Z,10Z-triene-8-ynoic acid isobutylamide, dodeca-2E,4E,10E-triene-8-ynoic acid isobutylamide, undeca-2E,4Z-diene-8,10-diynoic acid isobutylamide, undeca-2E/Z-ene-8,10-diynoic acid isobutylamide, dodeca-2E,4Z-diene-8,10-diynoic acid isobutylamide, dodeca-2E-ene-8,10-diynoic acid isobutylamide, trideca-2E,7Z-diene-10,12-diynoic acid isobutylamide, dodeca-2,4-diene-8,10-diynoic acid 2-methylbutylamide, and dodeca-2E,4Z,10E-triene-8-ynoic acid 2-methylbutylamide or dodeca-2E-ene-8,10-diynoic acid 2-methylbutylamide; (ii) DE-F-F2 ii constituted by dodeca-2,4,10-triene-8-ynoic acid isobutylamide isomer 1, trideca-2E,7Z-diene-10,12-diynoic acid 2-methylbutylamide, dodeca-2E,4E,8Z,10E/Z-tetraenoic acid isobutylamide, and pentadeca-2E,9Z-diene-12,14-diynoic acid isobutylamide; and (iii) DE-F-F2 iii that presented dodeca-2E,4E,8Z-trienoic acid isobutylamide isomer 1, dodeca-2E,4E,8Z-trienoic acid isobutylamide, dodeca-2E,4E,8Z,10E/Z-tetraenoic acid 2-methylbutylamide, and dodeca-2E,4E-dienoic acid isobutylamide. It is important to highlight that none of the alkylamides was repeated in each sub-fraction of DE-F-F2, empathizing the efficiency of the separation method.

EF-F1 was composed of protocatechuic acid, chlorogenic acid, caffeic acid, chicoric acid, rutin, and rutin derivative. EF-F2 was constituted by dodeca-2E,4Z,10E-triene-8-ynoic acid isobutylamide, dodeca-2E,4Z,10Z-triene-8-ynoic acid isobutylamide, dodeca-2E,4E,10Z-triene-8-ynoic acid isobutylamide, dodeca-2E,4E,10E-triene-8-ynoic acid isobutylamide, undeca-2E,4Z-diene-8,10-diynoic acid isobutylamide, undeca-2E/Z-ene-8,10-diynoic acid isobutylamide, pentadeca-2E,9Z-diene-12,14-diynoic acid 2-hydroxyisobutylamide, dodeca-2E,4Z-diene-8,10-diynoic acid isobutylamide, dodeca-2E-ene-8,10-diynoic acid isobutylamide, trideca-2E,7Z-diene-10,12-diynoic acid isobutylamide, dodeca-2,4-diene-8,10-diynoic acid 2-methylbutylamide, trideca-2E,7Z-diene-10,12-diynoic acid 2-methylbutylamide, dodeca-2E,4E,8Z,10E/Z-tetraenoic acid isobutylamide, dodeca-2E,4Z,10E-triene-8-ynoic acid 2-methylbutylamide or dodeca-2E-ene-8,10-diynoic acid 2-methylbutylamide, pentadeca-2E,9Z-diene-12,14-diynoic acid isobutylamide, dodeca-2E,4E,8Z-trienoic acid isobutylamide, dodeca-2E,4E,8Z,10E/Z-tetraenoic acid 2-methylbutylamide, and dodeca-2E,4E-dienoic acid isobutylamide.

### 3.3. Cytotoxicity of E. purpurea Extracts and Fractions

**Figure 4:**
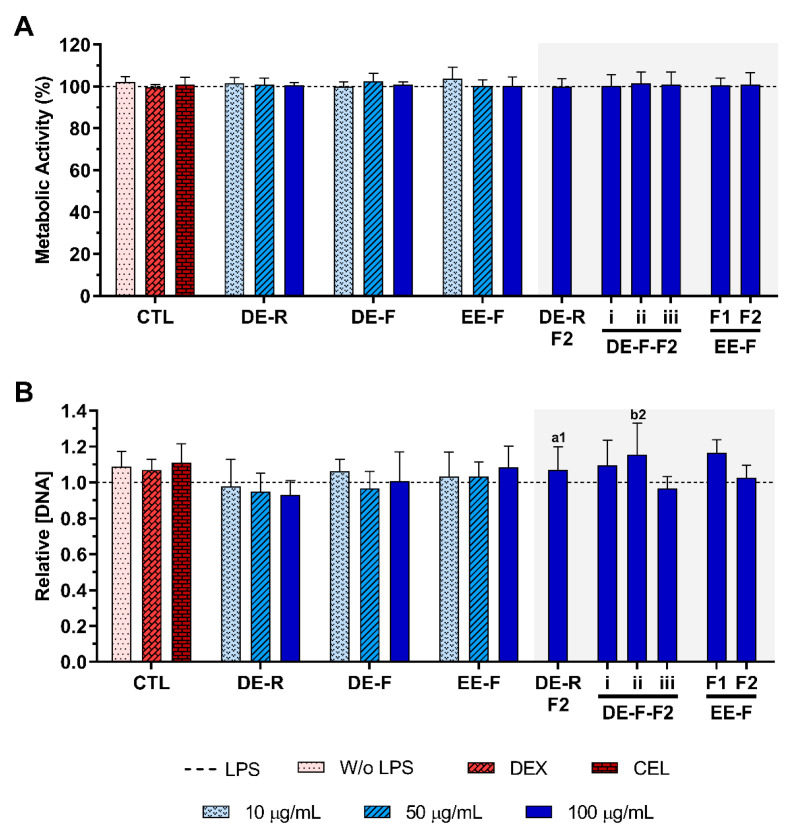
Metabolic activity (**A**) and relative DNA concentration (**B**) of LPS-stimulated human monocyte-derived macrophages (hMDMs) cultured in the presence of different concentrations of the *E. purpurea* extracts, fractions, and clinically used anti-inflammatory drugs (dexamethasone (DEX) and celecoxib (CEL), 10 μM) for 24 h. The dotted line represents the metabolic activity and DNA concentration of positive control (LPS-stimulated hMDM without treatment). There are no statistically significant differences in comparison to the positive control for each tested extract, fraction, DEX, and CEL. Statistically significant differences are 1 (*p* < 0.0133) and 2 (*p* < 0.0035) in comparison with a (DE-R vs. DE-R-F2) and b (DE-F vs. DE-F-F2) at the same concentration. CTL: control; DE: dichloromethanolic extracts; EE: ethanolic extracts; R: roots; F: flowers; F1: phenol/-carboxylic acid fraction; F2: alkylamide fraction.

The metabolic activity and the relative DNA concentration of LPS-stimulated hMDM in the absence or presence of *E. purpurea* extracts and fractions at different concentrations are presented in Figure 4. The cell metabolic activity and the DNA concentration were not affected by the presence of the DE, EE, and fractions at any tested concentration (Figure 4).

### 3.4. Anti-Inflammatory Activity of E. purpurea Extracts and Fractions

#### 3.4.1. Cytokine Production

**Figure 5:**
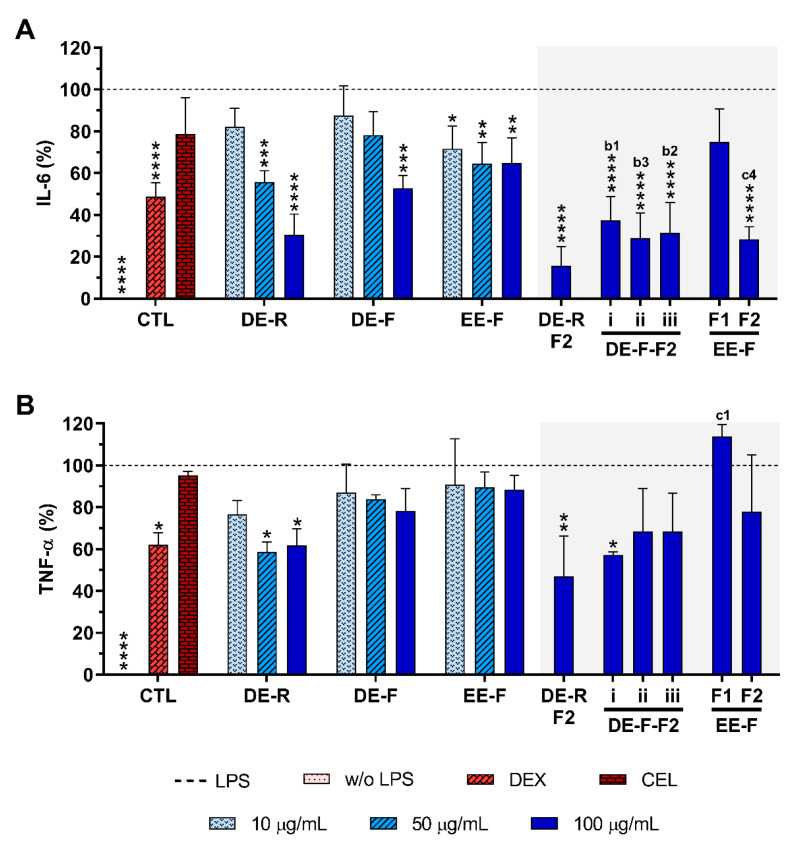
IL-6 (**A**) and TNF-α (**B**) production by LPS-stimulated human monocyte-derived macrophages (hMDMs) cultured in the presence of different concentrations of the *E. purpurea* extracts, fractions, and clinically used anti-inflammatory drugs (dexamethasone (DEX) and celecoxib (CEL), 10 μM) for 24 h. Statistically significant differences are * (*p* < 0.0363), ** (*p* < 0.0047), *** (*p* < 0.0003), and **** (*p* < 0.0001) in comparison to the positive control (LPS-stimulated hMDM without treatment) for all tested *E. purpurea* extracts, fractions, DEX, and CEL and 1 (*p* < 0.0428), 2 (*p* < 0.0018), 3 (*p* < 0.0003), and 4 (*p* < 0.0001) in comparison with b (DE-F vs. DE-R-F2) and c (EE-F vs. EE-F-F) at the same concentration. CTL: control; DE: dichloromethanolic extracts; EE: ethanolic extracts; R: roots; F: flowers; F1: phenol/carboxylic acid fraction; F2: alkylamide fraction.

The anti-inflammatory activity of *E. purpurea* extracts and fractions was evaluated by the decreased amounts of pro-inflammatory cytokines, namely IL-6 and TNF-α, in the cell culture supernatant of LPS-stimulated hMDM (Figure 5). Non-stimulated hMDM produced basal amounts of IL-6 (8.0 ± 10.5 pg/mL) and TNF-α (60.0 ± 36.0 pg/mL). As expected, LPS stimulation of hMDM led to a significant increase in the levels of these pro-inflammatory cytokines (IL-6: 19,139.0 ± 7850.8 pg/mL, TNF-α: 21,773.4 ± 9425.9 pg/mL). Dexamethasone (10 μM) effectively reduced the IL-6 and TNF-α production by 51.2 ± 6.5% and 38 ± 5.7%, respectively (Figure 5). As expected, celecoxib (10 μM) did not considerably decrease the IL-6 and TNF-α production (21.2 ± 17.2% and 4.6 ± 1.8%, respectively).

When LPS-stimulated hMDMs were treated with the whole *E. purpurea* extracts, a significant decrease in the IL-6 amount in the culture supernatant was observed in a concentration-dependent manner (Figure 5A). Particularly, DE showed a higher ability to decrease IL-6 and TNF-α levels than EE. Moreover, the extracts obtained from roots more significantly reduced these two pro-inflammatory cytokines in comparison with the ones obtained from flowers. Indeed, 50 μg/mL of DE-R efficiently decreased the IL-6 production, being even more effective at 100 μg/mL (69.5 ± 10.0%). DE-F was only able to significantly decrease the IL-6 levels by 47.3 ± 6.2% at 100 μg/mL. A comparable significant IL-6 reduction was observed for EE-F over all tested concentrations, with the highest tested concentration displaying greater activity (35.2 ± 12.1%). DE-R was ≈1.5 and 2 times stronger than DE-F and EE-F, respectively. As extracts, all the fractions strongly decreased the IL-6 production, except the EE-F-F1 (25.1 ± 15.8%). Moreover, the bioactivity of DE-F and EE-F was significantly improved with their fractionation into F2. DE-R-F2 led to a more marked IL-6 reduction (84.3 ± 9.1%). DE-F-F2 i, DE-F-F2 ii, DE-F-F2 iii, and EE-F-F2 demonstrated similar bioactivity (62.7 ± 11.5%, 71.2 ± 12.3%, 68.5 ± 14.3%, and 71.6 ± 6.1%, respectively). Besides DE-R-F2 exhibiting a higher efficacy in IL-6 reduction, no significant differences were observed. Analyzing all the *E. purpurea* extracts and fractions, DE-R-F2 led to the strongest reduction in IL-6 production, followed by EE-F-F2 ≈ DE-F-F2 ii, DE-R ≈ DE-F-F2 iii, DE-F-F2 i, DE-F, EE-F, and EE-F-F1. Moreover, DE-R, DE-F, and all the F2 fractions demonstrated similar or higher bioactivity than dexamethasone.

Some extracts and fractions were also able to significantly decrease the TNF-α levels in LPS-stimulated hMDM cultures (Figure 5B). DE-R, at 50 μg/mL, significantly decreased the TNF-α production by 41.4 ± 4.9%. Conversely, DE-F and EE-F did not demonstrate the capacity to markedly decrease the TNF-α production (21.7 ± 10.5% and 11.6 ± 6.9%, respectively). DE-R was ≈1.7 and 3.3 times stronger than DE-F and EE-F, respectively. Regarding the fractions, only DE-R-F2 and DE-F-F2 i significantly reduced the TNF-α production by 53.1 ± 19.5% and 42.7 ± 1.4%, respectively. Indeed, the bioactivity of the DE-R and DE-F extracts was not significantly improved with their fractionation. DE-F-F2 ii, DE-F-F2 iii, and EE-F-F2 did not show an ability to significantly reduce the TNF-α production (31.6 ± 20.6%, 31.6 ± 18.2%, and 22.1 ± 27.0%, respectively). EE-F-F1 increased the TNF-α amount by 13.9 ± 5.7% in comparison to LPS-stimulated hMDM. Comparing the data obtained for all *E. purpurea* extracts and fractions, it is possible to conclude that DE-R-F2 exhibited the strongest reduction in TNF-α production, followed by DE-F-F2 i, DE-R, DE-F-F2 ii ≈ DE-F-F2 iii, EE-F-F2, DE-F, EE-F, and EE-F1. Moreover, DE-R, DE-R-F2, and DE-F-F2 demonstrated similar or higher bioactivity than dexamethasone.

#### 3.4.2. ROS/RNS/O_2_^•−^ Generation

**Figure 6:**
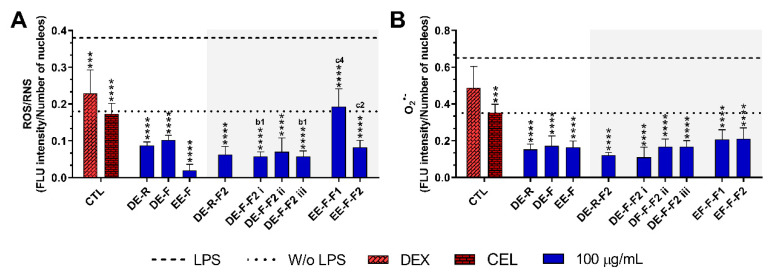
Fluorescence intensity of intracellular ROS/RNS (**A**) and O_2_^•−^ (**B**) production in LPS-stimulated human monocyte-derived macrophages (hMDMs) in the absence or presence of *E. purpurea* extracts, fractions, and clinically used anti-inflammatory drugs (dexamethasone (DEX) and celecoxib (CEL), 10 μM) cultured for 24 h. Fluorescence intensity was measured using ImageJ software. The dotted line represents the basal levels of ROS/RNS and O_2_^•−^ in non-stimulated hMDM (negative control) and the dashed line corresponds to the amounts of ROS/RNS and O_2_^•−^ produced by LPS-stimulated hMDM (positive control). Statistically significant differences are *** (*p* < 0.0002) and **** (*p* < 0.0001) in comparison to the positive control (LPS-stimulated hMDM without treatment) for each tested *E. purpurea* extract, fraction, DEX, and CEL, as well as 1 (*p* < 0.0453), 2 (*p* < 0.0023), and 4 (*p* < 0.0001) in comparison with b (DE-F vs. DE-R-F2) and c (EE-F vs. EE-F-F) at the same concentration. CTL: control; DE: dichloromethanolic extracts; EE: ethanolic extracts; R: roots; F: flowers; F1: phenol/carboxylic acid fraction; F2: alkylamide fraction.

The reduction in the intracellular levels of ROS/RNS and O_2_^•−^ in LPS-stimulated hMDM incubated with *E. purpurea* extracts and fractions at the maximal tested concentration are present in Figure 6 and Supplementary Figures S1 and S2. Non-stimulated hMDM produced basal levels of ROS and O_2_^•−^, which were significantly increased by the stimulation with LPS (Figure 6 and Supplementary Figure S1). Dexamethasone (10 μM) effectively reduced the ROS/RNS generation, but no differences were observed with the positive control in the reduction in O_2_^•−^ (Figure 6). Conversely, celecoxib (10 μM) considerably decreased both intracellular ROS/RNS and O_2_^•−^ generation.

The treatment of LPS-stimulated hMDM with the *E. purpurea* extracts drastically decreased the intracellular levels of ROS/RNS (Figure 6A and Supplementary Figure S2). EE-F demonstrated a strong capacity to decrease intracellular ROS/RNS production. EE-F was ≈4 and 5 times stronger than DE-R and DE-F, respectively. Regarding the fractions, all of them strongly decreased the intracellular ROS/RNS production. The fractionation of DE-F into DE-F-F2 i and DE-F-F2 iii significantly enhanced the reduction in intracellular ROS/RNS generation. However, the same trend was not observed for EE-F and its fractions. Indeed, the antioxidant activity was markedly decreased with the fractionation of EE-F into EE-F-F1 and EE-F-F2. DE-R-F2 and DE-F-F2 ii seem to present greater bioactivity than the whole extract, but no significant differences were observed. Analyzing all the *E. purpurea* extracts and fractions, EE-F demonstrated the most potent bioactivity, followed by DE-F-F2 i ≈ DE-F-F2 iii, DE-R-F2, DE-F-F2 ii, DE-R ≈ EE-F-F2, DE-F, and EE-F-F1. Moreover, all extracts and fractions were able to reestablish or decrease the levels to those observed in the non-stimulated hMDM.

As observed for ROS/RNS, the treatment of LPS-stimulated hMDM with the *E. purpurea* extracts significantly decreased the intracellular levels of O_2_^•-^ (Figure 6B and Supplementary Figure S2). The three extracts showed similar bioactivity in the reduction in O_2_^•−^ amounts. Additionally, all the fractions were also able to reduce the intracellular O_2_^•−^ a generation with comparable efficacy. Consequently, the fractionation of the extracts did not significantly improve their ability to reduce the intracellular O_2_^•−^ generation. Comparing all *E. purpurea* extracts and fractions, DE-F-F2 i exhibited the most powerful activity, followed by DE-R-F2, DE-R, EE-F, DE-F-F2 ii ≈ DE-F-F2 iii, DE-F, EE-F-F1, and EE-F-F2. Moreover, LPS-stimulated hMDM in the presence of all the extracts and fractions were able to reach similar or inferior levels of intracellular O_2_^•−^ to those observed in the non-stimulated macrophages.

#### 3.4.3. Therapeutic Targets

**Figure 7:**
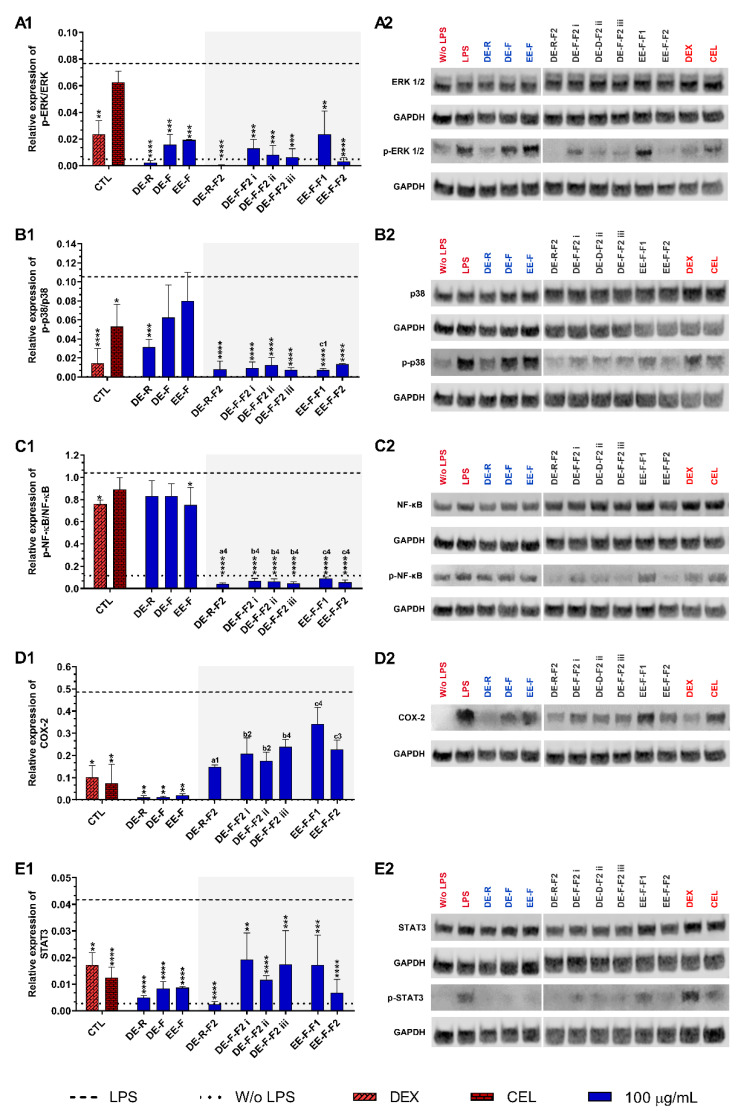
ERK 1/2 (**A1**,**A2**), p38 (**B1**,**B2**), NF-κB p65 (**C1**,**C2**), COX-2 (**D1**,**D2**), and STAT3 (**E1**,**E2**) signaling pathways downregulation of LPS-stimulated human monocyte-derived macrophages (hMDMs) cultured in the presence of *E. purpurea* extracts, factions, and clinically used anti-inflammatory drugs (dexamethasone (DEX) and celecoxib (CEL), 10 μM) for 24 h. Statistically significant differences are * (*p* < 0.0381), ** (*p* < 0.0071), *** (*p* < 0.0009), and **** (*p* < 0.0001) in comparison to the positive control (LPS-stimulated hMDM without treatment) for each tested *E. purpurea* extract and fraction, as well as DEX and CEL and 1 (*p* < 0.0358), 2 (*p* < 0.0023), 3 (*p* < 0.0002), and 4 (*p* < 0.0001) in comparison with a (DE-R vs. DE-R-F2), b (DE-F vs. DE-R-F2), and c (EE-F vs. EE-F-F) at the same concentration. CTL: control; DE: dichloromethanolic extracts; EE: ethanolic extracts; R: roots; F: flowers; F1: phenol/carboxylic acid fraction; F2: alkylamide fraction.

To understand the therapeutic targets responsible for the anti-inflammatory activity of the *E. purpurea* extracts and fractions, several pro-inflammatory signaling pathways were investigated by Western blot (Figure 7). Non-stimulated hMDM showed basal levels of ERK 1/2 (Figure 7A), p38 (Figure 7B), and NF-κB p65 phosphorylation (Figure 7C), but COX-2 (Figure 7D) and STAT3 (Figure 7E) expressions were not observed. The phosphorylation of all the studied inflammatory proteins was significantly enhanced in LPS-stimulated hMDM. Dexamethasone (10 µM) was able to significantly decrease the phosphorylation of all studied inflammatory proteins. Celecoxib (10 µM) also significantly reduced the phosphorylation of p38, STAT3, and the expression of COX-2, but no significant activity was observed for ERK 1/2 and NF-κB p65.

When LPS-stimulated hMDMs were treated with the whole *E. purpurea* extracts, a marked decrease in the activation of the ERK 1/2 signaling pathway was observed (Figure 7A). DE-R also efficiently decreased the phosphorylation of ERK 1/2, being its activity ≈8 and 9.5 times higher than DE-F and EE-F, respectively. As observed in extracts, the fractions reduced the phosphorylation of ERK 1/2, but DE-R-F2 and EE-F-F2 strongly suppressed the phosphorylation of this inflammatory protein. DE-F-F2 and EE-F-F1 demonstrated similar activity. Although F2 exhibited stronger bioactivity, no significant differences were observed compared to the whole extracts. Analyzing all the *E. purpurea* extracts and fractions, DE-R-F2 strongly suppressed the ERK 1/2 signaling pathway, followed by DE-R, EE-F-F2, DE-F-F2 iii, DE-F-F2 ii, DE-F-F2 i, DE-F, EE-F, and EE-F-F1. Moreover, LPS-stimulated hMDM in the presence of DE-R, DE-R-F2, DE-F-F2 ii, DE-F-F2 iii, and EE-F-F2 reached similar or lower levels of ERK 1/2 phosphorylation than non-stimulated macrophages.

Only DE-R was able to significantly reduce the activity of the p38 signaling pathway, being its bioactivity ≈2 and 2.7 times stronger than DE-F and EE-F, respectively (Figure 7B). DE-F and EE-F also led to decreased p38 phosphorylation, but no significant differences were observed. All the fractions significantly reduced the phosphorylation of p38, being DE-R-F2, DE-F-F2 iii, and EE-F-F1 the most promising. DE-F-F2 i, DE-F-F2 ii, and EE-F-F2 exhibited comparable bioactivity. Only the fractionation of EE-F into EE-F-F1 markedly reduced the p38 signaling pathway. Although other fractions presented increased bioactivity, no significant differences were observed in comparison with the whole extract. The comparison of all the *E. purpurea* extracts and fractions demonstrates a strong potential for DE-R-F2, DE-F-F2 iii, and EE-F-F1 in the reduction in p38 phosphorylation, followed by DE-F-F2 i ≈ DE-F-F2 ii ≈ EE-F-F2, DE-R, and DE-F ≈ EE-F.

Only EE-F was able to significantly reduce the activity of the NF-κB p65 signaling pathway, being its efficacy ≈1.1 times higher than DE-R and DE-F (Figure 7C). DE-F and EE-F showed a small ability to decrease the NF-κB p65 phosphorylation, with no significant differences. The fractionation of the whole *E. purpurea* extracts into fractions strongly improved the reduction in the NF-κB p65 signaling pathway. Indeed, all fractions markedly reduced the phosphorylation of NF-κB p65, being DE-R-F2, DE-F-F2 iii, and EE-F-F1 the most promising. DE-F-F2 i, DE-F-F2 ii, and EE-F-F2 demonstrated equivalent bioactivity. Comparing all the *E. purpurea* extracts and fractions, DE-R-F2, DE-F-F2 iii, and EE-F-F1 exhibited the most powerful bioactivity in the reduction in NF-κB p65 phosphorylation, followed by DE-F-F2 ii ≈ DE-F-F2 i ≈ EE-F-F1, EE-F, and DE-R ≈ DE-F. Moreover, all the fractions enabled LPS-stimulated hMDM to reach lower levels of ERK 1/2 phosphorylation than non-stimulated macrophages.

The treatment of LPS-stimulated hMDM with the whole *E. purpurea* extracts significantly suppressed the COX-2 expression (Figure 7D). Despite the similar bioactivity of the three extracts, DE-R showed to be ≈1.2 and 1.9 times stronger than DE-F and EE-F. The fractions decreased the COX-2 expression, but no significant differences were observed. Nevertheless, DE-R-F2, DE-F-F2, and EE-F-F2 presented comparable bioactivity, while EE-F-F1 showed a lower ability to reduce COX-2 expression. In this case, the fractionation of the whole *E. purpurea* extracts into fractions did not enhance the reduction in COX-2 expression by LPS-stimulated hMDM. Comparing all the *E. purpurea* extracts and fractions, DE-R and DE-F led to the most potent COX-2 suppression, followed by EE-F, DE-R-F2, DE-F-F2 ii, DE-F-F2 i, DE-F-F2 iii, EE-F-F2, DE-F-F2 iii, and EE-F-F1.

*E. purpurea* extracts also showed a strong capacity to reduce the activation of the STAT3 signaling pathway in LPS-stimulated hMDM (Figure 7E). DE-R efficiently decreased the phosphorylation of STAT3, being its activity ≈1.6 and 1.8 times higher than DE-F and EE-F, respectively. All the fractions also significantly reduced the phosphorylation of STAT3, but DE-R-F2, followed by EE-F-F2 and DE-F-F2 ii, strongly suppressed the phosphorylation of this inflammatory protein. DE-F-F2 i, DE-F-F2 iii, and EE-F-F1 presented less but similar bioactivity. Nevertheless, only DE-R-F2 and EE-F-F2 improved the bioactivity of the extracts, but no significant differences were observed. Analyzing all the *E. purpurea* extracts and fractions, DE-R-F2 was the most promising formulation in the reduction in STAT3 phosphorylation, followed by DE-R, EE-F-F2, DE-F, EE-F, DE-F-F2 ii, and DE-F-F2 iii ≈ EE-F-F1 ≈ DE-F-F2 i. Moreover, LPS-stimulated hMDM in the presence of DE-R and DE-R-F2 achieved similar or lower levels of STAT3 phosphorylation compared with non-stimulated macrophages.

The inflammatory proteins JNK/p-JNK and iNOS were also investigated, but no phosphorylation expression was detected in this study at 24 h of culture.

## 4. Discussion

The development of chronic pathologies, such as rheumatoid arthritis and osteoarthritis, is strongly correlated with persistent inflammation [12,13]. Additionally, most of the current treatments are associated with significant side effects, and thus, effective and safe therapies are urgently needed. As the initiation and progression of inflammation involve several inflammatory signaling pathways, new and safe entities that effectively modulate different molecular mechanisms are required.

This study used DE-R, DE-F, and EE-F since they exhibited the strongest anti-inflammatory properties in our previous work [42]. These three extracts presented different patterns of phenolic compounds, carboxylic acids, and alkylamides in their composition, comparable to our previous study [42]. Briefly, we identified a higher number of phenolic and carboxylic acids in EE-F (11 compounds) than in DE-R (4 compounds) or DE-F (3 compounds). Relative to the alkylamides, DE-R was the extract where more alkylamides were identified (24 compounds), followed by DE-F (20 compounds) and EE-F (19 compounds). As phenolic compounds, carboxylic acids, and alkylamides have different polarities, the extracts were fractionated into F1 (phenols/carboxylic acids) and F2 (alkylamides) fractions. Afterward, the anti-inflammatory activity of the cytocompatible extracts and fractions was investigated by their ability to decrease IL-6, TNF-α, and ROS/RNS levels in LPS-stimulated hMDM.

The three whole extracts drastically reduced the IL-6 levels in LPS-stimulated hMDM. This ability is important since IL-6 induces hematopoiesis, promotes the expansion and activation of T cells, stimulates B cell differentiation, and regulates neutrophil-activating chemokines, among other processes [6,11]. When the extracts were fractionated into F2, the anti-inflammatory activity was considerably enhanced. On the other hand, the F1 obtained from EE-F did not significantly reduce the IL-6 amount. Therefore, it is possible to conclude that alkylamides are the main class of compounds responsible for the decrease in the IL-6 production by LPS-stimulated hMDM. Moreover, the bioactivity was increased when a high number of alkylamides was present, which involves possible synergistic effects. Specifically, DE-R-F2, composed of 19 alkylamides, demonstrated greater IL-6 reduction (84.3 ± 9.1%) than DE-F-F2 i (10 alkylamides, 62.7 ± 11.5%), DE-F-F2 ii (4 alkylamides, 71.2 ± 12.3%), or DE-F-F2 iii (4 alkylamides, 68.5 ± 14.3%).

DE-R was the only extract that significantly decreased TNF-α levels (41.4 ± 4.9%). Although the fractionation of extracts into fractions enhanced the bioactivity of DE-R-F2 (53.1 ± 19.5%) and DE-F-F2 i (42.7 ± 1.4%), no significant differences between whole extract and fractions were observed. Conversely, EE-F-F1 failed to decrease TNF-α production, and, consequently, its ability to recruit and enhance the differentiation and proliferation of the immune cells, as well as induce the transcription of several inflammatory genes [52]. These results strengthen the role of alkylamides as promising anti-inflammatory candidates. Furthermore, the bioactive pattern and the decrease in IL-6 and TNF-α levels obtained for LPS-stimulated hMDM are in agreement with our previous study with LPS-stimulated THP-1-derived macrophages [42]. These results support the correlation and similar behavior between the human cell line and primary cells. Nonetheless, it is important to stress that the bioactivity of the extracts was slightly lower in primary macrophages.

All the studied extracts and fractions strongly reduced the intracellular levels of ROS/RNS/O_2_^•−^ in LPS-stimulated hMDM, reaching similar or inferior levels to those of the non-stimulated macrophages. Particularly, the fractionated DE-F-F2 i and DE-F-F2 ii significantly reduced the intracellular ROS/RNS generation, suggesting that the alkylamides present in these fractions are directly involved in this bioactivity. Moreover, alkylamide fractions showed, in general, lower intracellular levels of ROS/RNS/O_2_^•−^, suggesting that these compounds may be the main compounds responsible for this bioactivity. In this study, the fractionation of EE-F into EE-F-F1 dramatically diminished the capacity of intracellular ROS/RNS reduction, besides phenols/carboxylic acids present in *E. purpurea* extracts are considered strong antioxidants in in vitro assays [53,54]. These results are in agreement with our previous study, where *E. purpurea* extracts enriched in alkylamides presented the strongest intracellular ROS/RNS reduction [42]. It can be hypothesized that alkylamides may inhibit the direct production, mainly in the mitochondria, of these inflammatory mediators [55,56,57]. Alkylamides may also interfere with the transcription of antioxidant enzymes, such as superoxide dismutase (SOD), catalase (CAT), and glutathione peroxidase (GPx) [58], as well as target the nitric oxide synthases (NOS) [59].

Different patterns were observed for the suppression of the inflammatory signaling pathways, demonstrating that extracts and fractions modulate different inflammatory mechanisms, including ERK 1/2, p38, NF-κB p65, COX-2, and STAT3, to diverse extents. The alkylamide fractions demonstrated stronger potential to drastically inhibit the inflammatory pathways, pointing out the main role of alkylamides in reducing inflammation. The synergistic effect between alkylamides was also observed since DE-R-F2 demonstrated strong bioactivity in general. We also demonstrated the downregulation of COX-2 expression in the presence of extracts, but not of fractions, in LPS-stimulated hMDM. Thus, a synergistic effect between the classes of compounds present in the whole extract should be required for the inhibitory effect of COX-2 expression.

The activation of the STAT3 signaling pathway in LPS-stimulated hMDM was strongly suppressed by extracts. DE-R-F2 was the only fraction that demonstrated higher bioactivity than the whole extract; however, no significant differences were observed. Nevertheless, once again, the alkylamides were the main compounds responsible for the reduction in this inflammatory pathway in LPS-stimulated hMDM. In this study, it was not possible to observe the inflammatory proteins p-JNK and iNOS, perhaps due to the occurrence of their expression at early time points [27,40].

The anti-inflammatory activity of *E. purpurea* preparations has been reported to be due to different alkylamides. Indeed, the major alkylamide found in *E. purpurea*, dodeca-2E,4E,8Z,10Z-tetraenoic acid isobutylamide, demonstrated minor anti-inflammatory effect compared to the alkylamide fraction [60]. Similarly to our results, Hou et al. reported that isolated chicoric acid did not show strong effects in the reduction in TNF-α levels in LPS-stimulated macrophages [27]. On the other hand, the isolated dodeca-2E,4E,8Z,10E/Z-tetraenoic acid isobutylamide potentially decreased the expression of this protein in LPS-stimulated primary human monocyte/macrophage-enriched PBMCs [61]. In fact, as previously reported, alkylamide fractions led to the robust inhibition of ^•^NO production in LPS-stimulated RAW 264.7 macrophages [27,40,60]. Indeed, alkylamides, including dodeca-2E,4Z-diene-8,10-diynoic acid isobutylamide (present in DE-R-F2, DE-F-F2 i, and EE-F-F2), dodeca-2E,4E,8Z,10E/Z-tetraenoic acid isobutylamide (present in DE-R-F2, DE-F-F2 ii, and EE-F-F2), dodeca-2E,4E-dienoic acid isobutylamide (present in DE-R-F2, DE-F-F2 iii, and EE-F-F2), dodeca-2E,4Z,10Z-triene-8-ynoic acid isobutylamide (present in DE-R-F2, DE-F-F2 i, and EE-F-F2), dodeca-2E,4E,8Z-trienoic acid isobutylamide (present in DE-R-F2, DE-F-F2 iii, and EE-F-F2), and undeca-2Z,4E-diene-8,10-diynoic acid isobutylamide (present in DE-R-F2), decreased the ^•^NO production in RAW 264.7 macrophages [29]. To the best of our knowledge, only isolated phenols obtained from *E. purpurea* have been reported to have an anti-inflammatory effect. Chicoric acid was able to decrease the TNF-α, IL-1β, and IL-6 levels and the infiltration of inflammatory cells in streptozotocin (STZ)-induced diabetic C57BL/6J mice [24,37]. MTX-induced liver injury or chronic kidney disease in male Wistar rats pre-treated with chicoric acid reduced TNF-α, ROS, ^•^NO, and malondialdehyde (MDA) levels [62,63].

Our results, together with the currently available evidence, suggest that alkylamides are powerful plant-based drugs, exhibiting strong pharmaceutical advantages to ameliorate the inflammatory process related to chronic diseases.

## 5. Conclusions

*E. purpurea* extracts efficiently decreased pro-inflammatory mediators (IL-6, TNF-α, and/or ROS/RNS) in LPS-stimulated hMDM, corroborating their anti-inflammatory effects. The fractionation of the whole extracts into alkylamide fractions drastically enhanced the bioactivity, evidencing these compounds as the main active principles. This study also showed that the combination of different phytochemical compounds exhibited high pharmacological properties. Particularly, an increased number of alkylamides demonstrated greater bioactivity. Moreover, alkylamides exert their anti-inflammatory activity through the reduction in ERK1/2, p38, NF-κB, and STAT3 inflammatory signaling pathways, and the downregulation of COX-2 expression. Therefore, *E. purpurea* extracts and fractions can revert and stop the hyperactivation of macrophages, reaching the desired homeostasis in chronic diseases and preventing damage of the surrounding cells and tissues. Consequently, these results point out the efficiency of *E. purpurea* extracts and fractions, particularly DE-R-F2, an alkylamide extract, as new, innovative, and powerful plant-based anti-inflammatory formulations in the modulation of the fate of macrophages in cases where the immune system is overactive. To the best of our knowledge, the anti-inflammatory activity of DE and DE fractions are studied here for the first time in LPS-stimulated hMDM, in which their therapeutic targets are reported. As the immune response involves both specific and non-specific mechanisms, further studies supporting the role of *E. purpurea* extracts and fractions in complex models of inflammation should be explored.

## Figures and Tables

**Table 1:** Parameters of the optimized gradient method for semi-preparative HPLC.

Time (Min)	Water with 0.1% Formic Acid (%)	ACN (%)
0	50	50
7	5	95
20	5	95
21	50	50
25	50	50

**Table 2:** Overview of the identified compounds (phenols/carboxylic acids and alkylamides) in *E. purpurea* extracts and fractions by LC-HRMS.

Compounds	DE		EE	DE-R	DE-F			EE-F	
	R	F	F	F2	F2 i	F2 ii	F2 iii	F1	F2
Malic Acid	+	-	+	-	-	-	-	-	-
Vanillic acid	-	-	+	-	-	-	-	-	-
Protocatechuic acid	-	-	+	-	-	-	-	+	-
Caftaric acid ^a^	-	-	+	-	-	-	-	-	-
Chlorogenic acid ^a^	-	-	+	-	-	-	-	+	-
Quinic acid	-	-	-	-	-	-	-	-	-
Vanillin	-	-	-	-	-	-	-	-	-
Caffeic acid ^a^	+	+	+	-	-	-	-	+	-
Benzoic acid	+	+	+	-	-	-	-	-	-
Cynarin ^a^	-	-	-	-	-	-	-	-	-
Echinacoside ^a^	-	-	-	-	-	-	-	-	-
*p*-coumaric acid	+	-	+	-	-	-	-	-	-
Chicoric acid ^a^	-	+	+	-	-	-	-	+	-
Rutin	-	-	+	-	-	-	-	+	-
Quercetin	-	-	+	-	-	-	-	-	-
Dodeca-2E,4Z,10E-triene-8-ynoic acid isobutylamide	+	+	+	+	+	-	-	-	+
Dodeca-2E,4Z,10Z-triene-8-ynoic acid isobutylamide	+	+	+	+	+	-	-	-	+
Dodeca-2,4,10-triene-8-ynoic acid isobutylamide (isomer 1)	+	+	-	-	-	+	-	-	-
Dodeca-2E,4E,10Z-triene-8-ynoic acid isobutylamide	+	+	+	+	-	-	-	-	+
Dodeca-2Z,4E,10Z-triene-8-ynoic acid isobutylamide	+	-	-	+	-	-	-	-	-
Dodeca-2E,4E,10E-triene-8-ynoic acid isobutylamide	+	+	+	+	+	-	-	-	+
Undeca-2E,4Z-diene-8,10-diynoic acid isobutylamide	+	+	+	+	+	-	-	-	+
Undeca-2E/Z-ene-8,10-diynoic acid isobutylamide^a^	-	+	+	-	+	-	-	-	+
Undeca-2Z,4E-diene-8,10-diynoic acid isobutylamide	+	-	-	+	-	-	-	-	-
Undeca-2E/Z,4Z/E-diene-8,10-diynoic acid 2-methylbutylamide	-	-	-	-	-	-	-	-	-
Pentadeca-2E,9Z-diene-12,14-diynoic acid 2-hydroxyisobutylamide	-	+	+	-	-	-	-	-	+
Dodeca-2E,4Z-diene-8,10-diynoic acid isobutylamide	+	+	+	+	+	-	-	-	+
Undeca-2E,4E-diene-8,10-diynoic acid isobutylamide	+	-	-	-	-	-	-	-	-
Dodeca-2Z,4E-diene-8,10-diynoic acid isobutylamide	-	-	-	-	-	-	-	-	-
Dodeca-2E-ene-8,10-diynoic acid isobutylamide ^a^	+	+	+	+	+	-	-	-	+
Trideca-2E,7Z-diene-10,12-diynoic acid isobutylamide	+	+	+	+	+	-	-	-	+
Dodeca-2,4-diene-8,10-diynoic acid 2-methylbutylamide	+	+	+	+	+	-	-	-	+
Dodeca-2Z,4Z,10Z-triene-8-ynoic acid isobutylamide	+	-	-	+	-	-	-	-	-
Trideca-2E,7Z-diene-10,12-diynoic acid 2-methylbutylamide	+	+	+	+	-	+	-	-	+
Dodeca-2E,4E,8Z,10E/Z-tetraenoic acid isobutylamide ^a^	+	+	+	+	-	+	-	-	+
Dodeca-2E,4Z,10E-triene-8-ynoic acid 2-methylbutylamide OR Dodeca-2E-ene-8,10-diynoic acid 2-methylbutylamide	+	+	+	+	+	-	-	-	+
Dodeca-2E,4E,8Z-trienoic acid isobutylamide (isomer 1)	-	+	+	-	-	-	+	-	-
Dodeca-2E,4E-dienoic acid isobutylamide (isomer 1)	-	-	-	-	-	-	-	-	-
Pentadeca-2E,9Z-diene-12,14-diynoic acid isobutylamide	+	+	+	+	-	+	-	-	+
Dodeca-2E,4E,8Z-trienoic acid isobutylamide	+	+	+	+	-	-	+	-	+
Trideca-2Z,7Z-diene-10,12-diynoic acid 2-methylbutylamide	+	-	-	-	-	-	-	-	-
Dodeca-2E,4E,8Z,10E/Z-tetraenoic acid 2-methylbutylamide	+	+	+	+	-	-	+	-	+
Hexadeca-2E,9Z-diene-12,14-diynoic acid isobutylamide	+	-	-	-	-	-	-	-	-
Dodeca-2E,4E,8Z-trienoic acid isobutylamide (isomer 2)	+	-	-	-	-	-	-	-	-
Dodeca-2E,4E-dienoic acid isobutylamide ^a^	+	+	+	+	-	-	+	-	+

DE: dichloromethanolic extracts; EE: ethanolic extracts; R: roots; F: flowers; F1: phenol/carboxylic acid fraction; F2: alkylamide fraction; symbol “+” represents the presence of compound; symbol “-” represents the absence of compound. ^a^ Injected standards. E/Z stereochemistry is indicated here in accordance to existing literature [44,45,46,47,48,49,50], but it should be highlighted that without NMR spectra, it is not possible to conclusively distinguish between E and Z isomers.

## Data Availability

The data presented in this study are available in the article and supplementary materials.

## Supplementary Materials

The following supporting information can be downloaded at: https://www.mdpi.com/article/10.3390/antiox12020425/s1. Figure S1: Intracellular ROS/RNS and O_2_^•−^ production in LPS-stimulated human monocyte-derived macrophages in the absence or in the presence of clinically used anti-inflammatory drugs; Figure S2: Intracellular ROS/RNS and O_2_^•−^ production in LPS-stimulated human monocyte-derived macrophages in the presence of *E. purpurea* extracts or fractions for 22 h.; Table S1: Parameters of the optimized gradient method for analytical separation; Table S2: Properties of standards determined by LC-HRMS.; Table S3: Phenolic/carboxylic acidic compounds tentatively identified in *E. purpurea* fractions by LC-HRMS (negative mode).; Table S4: Alkylamides compounds tentatively identified in *E. purpurea* fractions by LC-HRMS (positive mode).
