# Supplementary material for: Echinacea purpurea Fractions Represent Promising Plant-Based Anti-Inflammatory Formulations
Source: Antioxidants (Basel). 2023 Feb 9;12(2):425. doi: 10.3390/antiox12020425 (PMC9952182; doi:10.3390/antiox12020425)
Supplement: Supplementary file 1 [file antioxidants-12-00425-s001.zip › antioxidants-2195102-supplementary.pdf]

Supplementary Material

Table S1. Parameters of the optimized gradient method for analytical separation.

Time (min)	Water with 0.1% formic acid (%)	ACN (%)
0	90	10
18	85	15
30	10	90
35	10	90
36	90	10
45	90	10

Table S2. Properties of caftaric acid, chlorogenic acid, caffeic acid, cynarin, echinacoside, chicoric acid, undeca-2E/Z-ene-8,10-diynoic acid isobutylamide, dode-ca-2E-ene-8,10-diynoic acid isobutylamide, dodeca-2E,4E-dienoic acid isobutylamide, and dodeca-2E,4E,8Z,10E/Z-tetraenoic acid isobutylamide determined by LC-HRMS.

Standard compound	Chemical structure	Formula	Exact mass	Theoretical precursor ion (*m*/*z*)	Precursor ion (*m*/*z*)	t_R (min)	Product ions (*m*/*z*)
Caftaric acid	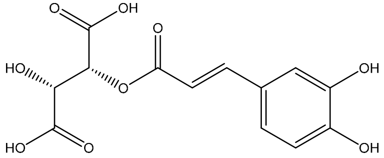	C13H12O9	312.04813196	311.0403	311.0405 $[M-H]^-$	3.1	135.0451 (100), 179.0350 (27), 149.0090 (17)
Chlorogenic acid	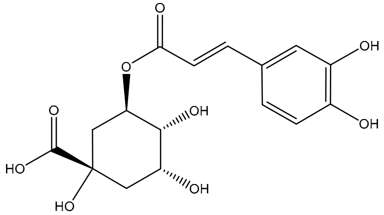	C16H18O9	354.09508215	353.0873	353.0874 $[M-H]^-$	4.1	191.0560 (100), 192.0592 (6), 161.0245 (2), 127.0401 (2), 85.0291 (2), 179.0346 (1)
Caffeic acid	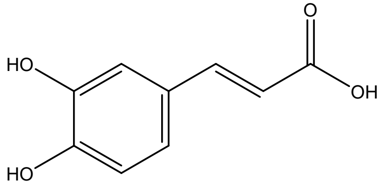	C9H8O4	180.04225873	179.0345	179.0350 $[M-H]^-$	5.1	135.0449 (100), 134.0372 (90), 89.0395 (9), 117.0343 (7), 107.0500 (7), 136.0484 (7)
Cynarin	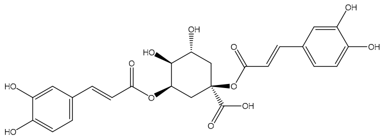	C25H24O12	516.12677620	515.1190	515.1195 $[M-H]^-$	6.2	191.0563 (100), 179.0351 (89), 353.0877 (22), 135.0453 (15), 180.0384 (8), 161.0245 (8), 192.0598 (7), 173.0459 (6), 354.0910 (4), 335.0768 (4)
Echinacoside	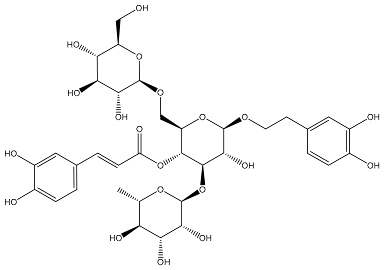	C35H46O20	786.25824385	785.2504	785.2505 $[M-H]^-$	7.3	785.2507 (100), 161.0246 (81), 623.2193 (64), 786.2539 (41), 624.2229 (18), 787.2581 (11), 162.0280 (8), 179.0357 (6)

Chicoric acid	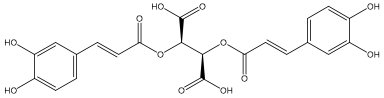	C22H18O12	474.07982601	473.0720	473.0725 $[M-H]^-$	9.0	179.0351 (100), 149.0094 (71), 135.0452 (17), 219.0299 (12), 180.0386 (7), 161.0243 (4), 293.0301 (4), 112.9881 (4), 311.0393 (1)
Undeca-2E/Z-ene-8,10-diynoic acid isobutylamide	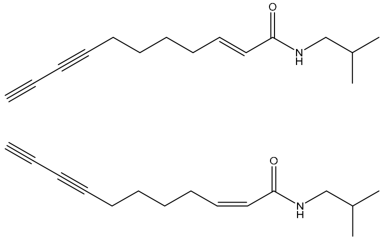	C15H21NO	231.16231	232.1701	232.1698 $[M-H]^+$	20.5	91.0544 (100), 115.0544 (43), 105.0701 (43), 116.0618 (35), 103.0544 (20), 129.0699 (12)
Dodeca-2E-ene-8,10-diynoic acid isobutylamide	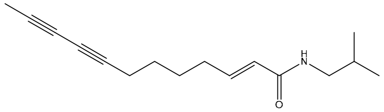	C16H23NO	245.17796	246.1858	246.1855 $[M-H]^+$	20.8	91.0544 (100), 105.0700 (83), 117.0699 (45), 115.0544 (36), 103.0544 (28), 129.0697 (24), 130.0774 (24), 77.0378 (20)
Dodeca-2E,4E,8Z,10E/Z-tetraenoic acid iso-butylamide	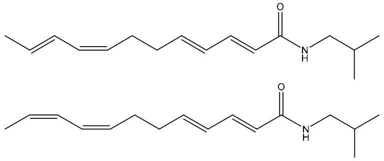	C16H25NO	247.19361	248.2014	248.2010 $[M-H]^+$	21.3	152.1071 (100), 95.0492 (59), 96.0447 (57), 110.0601 (18), 124.0758 (14), 167.1303 (13), 91.0543 (10), 112.0758 (10), 153.1103 (10), 81.0691 (9)
Dodeca-2E,4E-dienoic acid isobu-tylamide	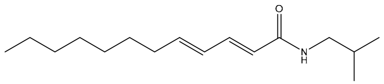	C16H29NO	251.22491	252.2327	252.2326 $[M-H]^+$	22.0	95.0856 (100), 98.0602 (93), 95.0493 (52), 81.0691 (43), 196.1696 (32), 112.0759 (28), 109.1013 (26), 126.0914(25)

Table S3. Phenolic/carboxylic acids compounds tentatively identified in *E. purpurea* fractions by LC-HRMS (negative mode).

Proposed phenolic compound	Precursor ion $[M-H]^-$ (m/z)	t_R (min)	Product ions (m/z) [1]
**Ethanolic Extracts obtained from Flowers – Faction 1 (EE-F-F1)**			
Protocatechuic acid	153.0195	2.8	108.0218 (100), 109.0287 (91), 91.0185 (17)
Chlorogenic acid[a]	353.0876	4.0	191.0562 (100), 192.0606 (5), 127.0404 (3), 85.0292 (2), 161.0250 (2), 179.0929 (0.3)

Caffeic acid[a]	179.0347	5.1	134.0373 (100), 135.0450 (92), 89.0395 (13), 136.0489 (9), 107.0498 (9), 117.0347 (7)
Chicoric acid[a]	473.0722	8.9	179.0349 (100), 149.0093 (70), 135.0452 (15), 219.0299 (13), 180.0384 (9), 161.0244 (4), 293.0299 (4), 112.9882 (3), 311.0420 (1)
Rutin	609.1459	9.2	300.0272 (100), 609.1457 (60), 301.0336 (44), 610.1491 (19), 302.0368 (7), 611.1523 (3)
Rutin derivative	609.1459	9.4	300.0273 (100), 609.1456 (63), 301.0341 (63), 610.1494 (19), 302.0372 (8), 611.1511 (4)

F: flowers; EE: ethanolic extracts; F1: phenol/-carboxylic acid fraction..

[a] Injected standards

[1] The MS^2 data were obtained from the fragmentation of the $[M - H]^-$ precursor ion of phenolic compounds. Relative intensities of product ions are in parentheses.

Table S4. Alkylamides compounds tentatively identified in *E. purpurea* fractions by LC-HRMS (positive mode).

Proposed alkylamide compound	Precursor ion $[M + H]^+$ (*m/z*)	t_R (min)	Product ions (*m/z*) [1]
**Dichloromethanolic Extracts obtained from Roots – Fraction 2 (DE-R-F2)**			
Dodeca-2E,4Z,10E-triene-8-ynoic acid isobutylamide	246.1852	14.9	145.1015 (46), 119.0860 (38), 128.0633 (11), 173.0948 (8), 93.0708 (6)
Dodeca-2E,4Z,10Z-triene-8-ynoic acid isobutylamide	246.1854	15.5	119.085 (53), 145.1008 (46), 93.0691 (11), 173.0947 (11), 128.0628 (9), 102.0925 (6)
Dodeca-2E,4E,10Z-triene-8-ynoic acid isobutylamide	246.1852	19.0	145.1018 (39), 128.0619 (38), 119.0480 (31), 93.0699 (22), 154.1227 (6)
Dodeca-2Z,4E,10Z-triene-8-ynoic acid isobutylamide	246.1853	19.4	145.1015 (35), 119.0854 (23), 128.0620 (21), 173.0963 (7), 93.0702 (5), 154.0761 (2)
Dodeca-2E,4E,10E-triene-8-ynoic acid isobutylamide	246.1852	20.2	128.0618 (85), 119.0860 (21), 93.0703 (17), 145.1009 (14), 154.0631 (6), 102.0906 (4)
Undeca-2E,4Z-diene-8,10-diynoic acid isobutylamide	230.1549	20.3	128.0621 (100), 91.0543 (47), 116.0617 (28), 129.0684 (21), 103.0543 (9), 117.0645 (3), 102.0464 (2), 157.0650 (1), 174.0914 (1), 142.0417 (0.1), 202.1588 (0), 188.14390 (0), 74.0146 (0), 57.0683 (0)
Undeca-2Z,4E-diene-8,10-diynoic acid isobutylamide	230.1546	20.6	128.062 (100), 91.0543 (41), 129.0686 (26), 116.0617 (26), 103.0542 (9), 117.0595 (4), 102.0465 (2), 157.0648 (2), 174.0912 (1), 188.1432 (0.4), 142.0651 (0.2), 202.1586 (0.1)
Dodeca-2E,4Z-diene-8,10-diynoic acid isobutylamide	244.1712	20.7	129.0687 (27), 91.0543 (22), 116.0606 (8), 188.1070 (2), 156.0624 (1), 157.0648 (0.4), 174.0898 (0.2), 216.1747 (0.1), 88.1111 (0), 57.0687 (0)
Dodeca-2E-ene-8,10-diynoic acid isobutylamide[a]	246.1851	20.9	128.0621 (100), 115.0543 (42), 105.0700 (29), 91.0543 (28), 117.0699 (26), 129.0685 (25), 103.0543 (13), 130.0768 (13)

Trideca-2E,7Z-diene-10,12-diynoic acid isobutylamide	258.1864	21.0	128.0622 (100), 117.0699 (24), 103.0543 (8), 142.0769 (4), 131.0782 (1), 156.0618 (1), 102.0465 (1), 157.0646 (0.4), 230.1897 (0.1), 202.1223 (0.1), 216.1745 (0.1), 185.0840 (0.1), 74.0141 (0)
Dodeca-2,4-diene-8,10-diynoic acid 2-methylbutylamide	258.1864	21.0	117.0699 (24), 91.0543 (22), 105.0700 (21), 143.0855 (14), 116.0604 (7), 142.0769 (4), 171.0804 (3), 188.1070 (3), 230.1538 (0.1), 168.1313 (0.1), 202.1223 (0.1), 88.1113 (0.1), 156.1365 (0), 71.0844 (0)
Dodeca-2Z,4Z,10Z-triene-8-ynoic acid isobutylamide2	246.1853	21.2	93.0699 (4), 119.0856 (3), 128.0621 (3), 102.0914 (2), 145.0649 (1), 154.1181 (0.4), 173.0943 (0.1), 57.0685 (0.1), 190.1608 (0), 74.0136 (0)
Trideca-2E,7Z-diene-10,12-diynoic acid 2-methylbutylamide	272.2009	21.2	116.0606 (25), 157.0999 (3), 202.1225 (1), 185.0879 (1)
Dodeca-2E,4E,8Z,10E/Z-tetraenoic acid isobutylamide[a]	248.2017	21.4	152.1071 (100), 95.0492 (58), 96.0446 (57), 110.0601 (17), 124.0758 (15), 167.1302 (13), 91.0543 (11), 153.1103 (10), 112.0756 (10), 81.0691 (9)
Dodeca-2E,4Z,10E-triene-8-ynoic acid 2-methylbutylamide OR Dodeca-2E-ene-8,10-diynoic acid 2-methylbutylamide	260.2008	21.2	145.1012 (20), 119.0856 (17), 116.0611 (16), 173.0956 (3), 142.1228 (1), 190.1223 (1), 88.1112 (0.2), 71.7045 (0)
Pentadeca-2E,9Z-diene-12,14-diynoic acid isobutylamide	286.2166	21.5	102.0915 (1), 185.1317 (1), 230.1549 (0.3), 213.1161 (0.2)
Dodeca-2E,4E,8Z-trienoic acid isobutylamide	250.2166	21.7	97.0491 (4), 102.0914 (3), 168.1345 (2), 83.0360 (2), 154.1212 (2), 194.1534 (1), 177.1271 (1), 149.1325 (0.4), 57.0679 (0.1)
Dodeca-2E,4E,8Z,10E/Z-tetraenoic acid 2-methylbutylamide	262.2167	21.6	116.1072 (3), 147.1170 (3), 88.1113 (1), 175.1114 (1), 192.1386 (0.3), 71.0845 (0.1)
Dodeca-2E,4E-dienoic acid isobutylamide[a]	252.2323	22.0	95.0856 (100), 98.0601 (91), 95.0493 (52), 81.0691 (42), 196.1695 (32), 112.0758 (27), 109.1012 (27), 126.0915 (23)
**Dichloromethanolic Extracts obtained from Flowers – Fraction 2 i (DE-F-F2 i)**			
Dodeca-2E,4Z,10E-triene-8-ynoic acid isobutylamide	246.1854	14.9	145.1013 (78), 119.0856 (27), 93.0699 (14), 128.0622 (10), 173.0975 (7)
Dodeca-2E,4Z,10Z-triene-8-ynoic acid isobutylamide	246.1854	15.5	145.1015 (64), 119.0858 (46), 93.0704 (18), 128.0624 (8), 173.0974 (7), 190.1224 (4), 154.1233 (4)
Dodeca-2E,4E,10E-triene-8-ynoic acid isobutylamide	246.1856	20.2	119.0858 (23), 93.0703 (14), 145.1015 (4), 128.0628 (3), 173.0963 (1)
Undeca-2E,4Z-diene-8,10-diynoic acid isobutylamide	230.1553	20.2	128.0624 (100), 91.0545 (43), 116.0619 (27), 129.0684 (19), 103.0545 (9), 117.0646 (3), 102.0467 (2), 157.0652 (1), 174.0918 (1), 142.0733 (0.1), 188.1431 (0), 202.1565 (0), 74.0148 (0), 57.0688 (0)

Undeca-2E/Z-ene-8,10-diynoic acid isobutylamide[a]	232.1701	20.5	91.0546 (100), 128.0625 (59), 115.0546 (51), 116.0620 (39), 105.0702 (36), 129.0693 (21), 103.0546 (20)
Dodeca-2E,4Z-diene-8,10-diynoic acid isobutylamide	244.1703	20.7	91.0546 (34), 129.0688 (24), 116.0618 (19), 156.0634 (2), 157.0652 (1), 188.1076 (0.4), 174.0912 (0.4), 88.1112 (0.1), 216.1756 (0), 57.0687 (0)
Dodeca-2E-ene-8,10-diynoic acid isobutylamide[a]	246.1857	20.9	152.1073 (100), 96.0448 (74), 91.0545 (66), 95.0494 (62), 105.0702 (53), 117.0701 (40), 115.0546 (34), 130.0776 (22), 129.0697 (22), 103.0545 (15), 77.0379 (10)
Trideca-2E,7Z-diene-10,12-diynoic acid isobutylamide	258.1860	21.0	117.0701 (62), 128.0624 (60), 103.0546 (22), 142.0776 (18), 131.0853 (8), 157.1013 (2), 102.0467 (2), 156.0627 (1), 202.1229 (1), 185.0964 (1), 230.1907 (0.1), 216.1751 (0.1), 74.0138 (0)
Dodeca-2,4-diene-8,10-diynoic acid 2-methylbutylamide	258.1860	21.0	91.0546 (74), 117.0701 (62), 105.0702 (30), 116.0607 (19), 142.0776 (18), 143.0847 (7), 168.0815 (1), 156.0627 (1), 202.1229 (1), 171.0797 (0.4), 188.1436 (0.3), 230.1907 (0.1), 88.1124 (0)
Dodeca-2E,4Z,10E-triene-8-ynoic acid 2-methylbutylamide OR Dodeca-2E-ene-8,10-diynoic acid 2-methylbutylamide	260.2010	21.1	116.0608 (20), 142.0775 (17), 145.1014 (7), 119.0855 (5), 173.0836 (0.4), 190.1239 (0.1), 88.0301 (0)
**Dichloromethanolic Extracts obtained from Flowers – Fraction 2 ii (DE-F-F2 ii)**			
Dodeca-2,4,10-triene-8-ynoic acid isobutylamide (isomer 1)	246.1855	18.3	119.0861 (54), 145.1017 (50), 93.0699 (21), 128.0622 (19), 102.0917 (8)
Trideca-2E,7Z-diene-10,12-diynoic acid 2-methylbutylamide	272.2011	21.2	116.0609 (21), 157.1010 (3), 202.1229 (1), 185.0957 (1), 88.1116 (0.1)
Dodeca-2E,4E,8Z,10E/Z-tetraenoic acid isobutylamide[a]	248.2028	21.2	152.1073 (100), 95.0493 (58), 96.0447 (56), 110.0603 (17), 124.0759 (13), 167.1305 (12), 153.1106 (10), 112.0758 (10), 91.0544 (10), 81.0692 (9)
Pentadeca-2E,9Z-diene-12,14-diynoic acid isobutylamide	286.2166	21.5	102.0914 (5), 57.0687 (0.3), 185.1322 (0.2), 230.153 (0.1)
**Dichloromethanolic Extracts obtained from Flowers – Fraction 2 iii (DE-F-F2 iii)**			
Dodeca-2E,4E,8Z-trienoic acid isobutylamide (isomer 1)	250.2167	21.4	154.1228 (14), 149.1327 (8), 83.0849 (6), 97.1014 (5), 177.1274 (4), 168.1375 (4), 194.1537 (2), 102.0909 (1)
Dodeca-2E,4E,8Z-trienoic acid isobutylamide	250.2170	21.6	97.0494 (3), 102.0915 (3), 168.1347 (2), 83.0359 (2), 154.1214 (2), 194.1541 (1), 177.1277 (1), 149.1328 (0.4), 57.0684 (0.1)
Dodeca-2E,4E,8Z,10E/Z-tetraenoic acid 2-methylbutylamide	262.2168	21.5	116.1072 (3), 147.1171 (2), 88.1113 (1), 175.1120 (0.5), 192.1385 (0.2), 71.0848 (0.1)

Dodeca-2E,4E-dienoic acid isobutylamide[a]	252.2325	22.0	95.0857 (100), 98.0603 (90), 95.0494 (50), 81.0692 (45), 196.1697 (31), 109.1013 (27), 112.0758 (26), 126.0915 (23)
**Ethanolic Extracts obtained from Flowers – Fraction 2 (EE-F-F2)**			
Dodeca-2E,4Z,10E-triene-8-ynoic acid isobutylamide	246.1852	14.9	145.1015 (51), 119.0859 (44), 93.0683 (13), 128.0617 (8), 173.0981 (6)
Dodeca-2E,4Z,10Z-triene-8-ynoic acid isobutylamide	246.1854	15.4	145.101 (49), 119.0860 (39), 128.0618 (8), 173.0962 (7), 93.0690 (7), 190.1218 (3), 154.1242 (2)
Dodeca-2E,4E,10Z-triene-8-ynoic acid isobutylamide	246.1854	18.9	145.1014 (51), 119.0857 (49), 93.0701 (18), 128.0623 (16), 173.0952 (6), 190.1215 (2), 154.1219 (2), 102.0916 (1)
Dodeca-2E,4E,10E-triene-8-ynoic acid isobutylamide	246.1854	20.2	119.0858 (29), 93.0700 (15), 145.1019 (6), 128.0621 (6), 173.0965 (1)
Undeca-2E,4Z-diene-8,10-diynoic acid isobutylamide	230.1545	20.2	128.0622 (100), 91.0543 (42), 116.0617 (27), 129.0682 (19), 103.0542 (9), 117.0648 (3), 102.0464 (2), 157.0649 (1), 174.0914 (1), 142.0636 (0.1), 202.1588 (0), 188.1420 (0), 57.0686 (0)
Undeca-2E/Z-ene-8,10-diynoic acid isobutylamide[a]	232.1697	20.5	91.0542 (100), 128.0622 (81), 115.0543 (57), 116.0617 (41), 105.0699 (34), 129.0689 (26), 103.0543 (20)
Pentadeca-2E,9Z-diene-12,14-diynoic acid 2-hydroxyisobutyl-amide	302.2116	20.6	118.0743 (2), 185.1326 (2), 213.1283 (0.4), 90.0422 (0.2)
Dodeca-2E,4Z-diene-8,10-diynoic acid isobutylamide	244.1699	20.7	91.0543 (36), 129.0688 (24), 116.0616 (20), 156.0632 (2), 157.0656 (1), 174.0914 (0.3), 188.1074 (0.3), 88.1110 (0.1), 216.1386 (0), 57.0693 (0)
Dodeca-2E-ene-8,10-diynoic acid isobutylamide[a]	246.1855	20.9	152.1071 (100), 96.0446 (77), 91.0543 (70), 105.07 (58), 117.0698 (44), 115.0544 (41), 129.0696 (25), 130.0776 (25), 103.0544 (18), 77.0378 (10)
Trideca-2E,7Z-diene-10,12-diynoic acid isobutylamide	258.1856	20.9	117.0700 (55), 128.0623 (50), 103.0544 (21), 142.0776 (19), 131.0855 (6), 157.1012 (2), 102.0466 (2), 156.0826 (1), 202.1231 (1), 185.0964 (1), 230.1909 (0.1), 216.1747 (0.1), 74.0144 (0)
Dodeca-2,4-diene-8,10-diynoic acid 2-methylbutylamide	258.1856	20.9	91.0544 (68), 117.0700 (55), 105.0700 (20), 142.0776 (19), 116.0605 (18), 143.0842 (5), 168.0814 (1), 156.0826 (1), 202.1231 (1), 188.1435 (0.3), 171.0775 (0.2), 230.1909 (0.1), 88.0634 (0)
Trideca-2E,7Z-diene-10,12-diynoic acid 2-methylbutyla-mide	272.2010	21.2	116.0606 (19), 157.1012 (4), 202.1232 (1), 185.0964 (1), 88.1113 (0.2)
Dodeca-2E,4E,8Z,10E/Z-tetrae-noic acid isobutylamide[a]	248.2021	21.4	152.1073 (100), 95.0493 (60), 96.0447 (58), 110.0602 (17), 124.076 (14), 167.1305 (13), 153.1106 (11), 112.0757 (10), 91.0543 (10), 81.0692 (10)
Dodeca-2E,4Z,10E-triene-8-ynoic acid 2-methylbutylamide	260.2009	21.2	119.0857 (27), 145.1014 (23), 116.0616 (22), 142.0770 (8), 173.0961 (3), 190.1227 (2)

OR<br>Dodeca-2E-ene-8,10-diynoic acid 2-methylbutylamide			
Pentadeca-2E,9Z-diene-12,14-diynoic acid isobutylamide	286.2166	21.5	102.0918 (4), 185.1302 (1)
Dodeca-2E,4E,8Z-trienoic acid isobutylamide	250.2168	21.6	97.0490 (4), 102.0916 (3), 168.1348 (2), 83.0359 (2), 154.1219 (1), 194.1542 (1), 149.1332 (1), 177.1272 (0.4), 57.068 (0.1)
Dodeca-2E,4E,8Z,10E/Z-tetraenoic acid 2-methylbutylamide	262.2168	21.5	116.1071 (3), 147.1174 (2), 175.1124 (1), 88.1116 (1), 192.1385 (0.3)
Dodeca-2E,4E-dienoic acid isobutylamide[a]	252.2325	22.0	95.0857 (100), 98.0602 (92), 95.0493 (53), 81.0693 (43), 196.1699 (32), 112.0759 (28), 109.1013 (28), 126.0916 (25)

F: flowers; R: roots; EE: ethanolic extracts; DE: dichloromethanolic extracts; F2: alkylamide fraction.

[a] Injected standards

[1] The MS^2 data were obtained from the fragmentation of the $[M + H]^+$ precursor ion of alkylamides. Relative intensities of product ions are in parentheses.

E/Z stereochemistry is indicated here in accordance with literature [1–7], but it should be highlighted that without conformational NMR spectra, it is not possible to conclusively distinguish between E and Z isomers.

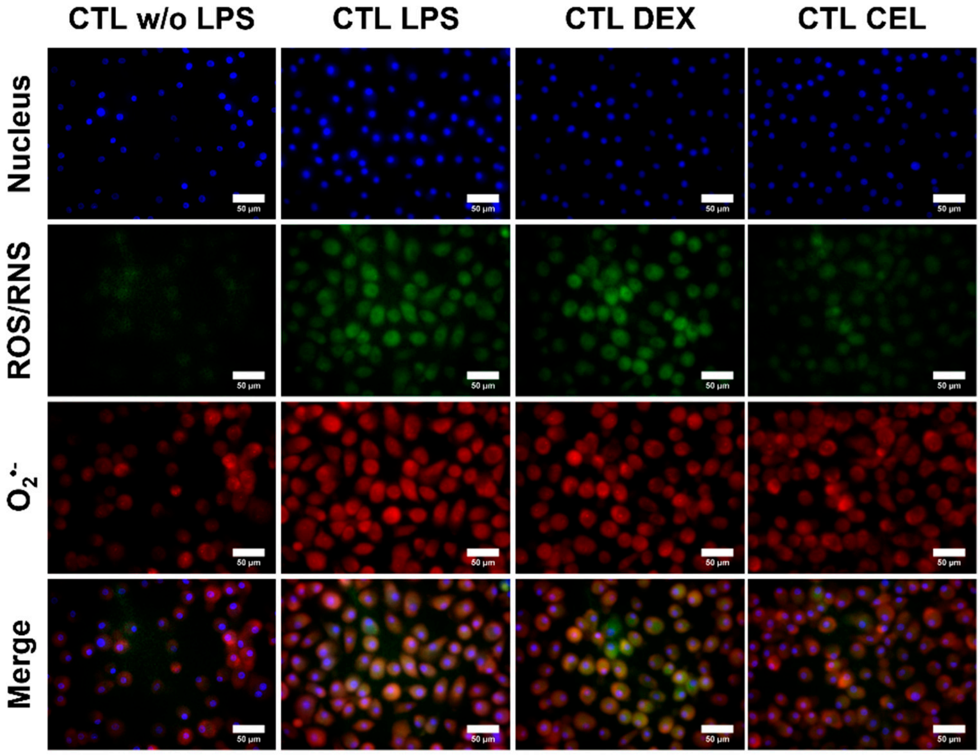

Figure S1. Intracellular ROS/RNS (green) and $O_2^{\bullet-}$ (red) production in LPS-stimulated human monocyte-derived macrophages (hMDM, nucleus in blue) in the absence or in the presence of clinically used anti-inflammatory drugs (dexamethasone -DEX- and celecoxib -CEL, 10 µM) for 24 h. CTL: control. Scale bar = 50 µm.

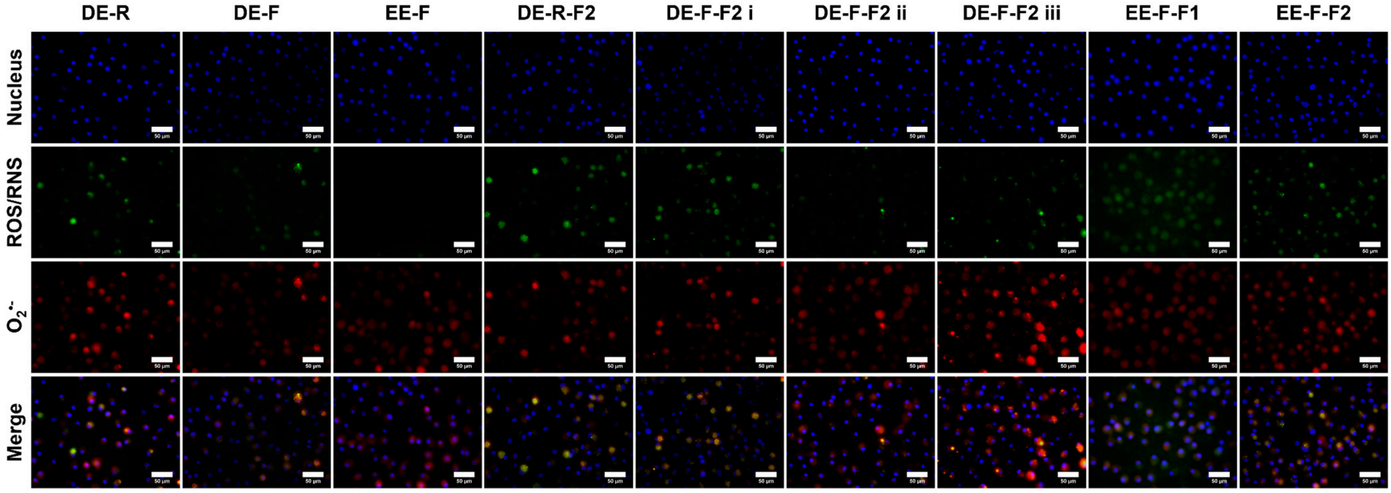

Figure S2. Intracellular ROS/RNS (green) and $O_2^{\bullet-}$ (red) production in LPS-stimulated human monocyte-derived macrophages (hMDM, nucleus in blue) in the presence of *E. purpurea* extracts or fractions for 22 h. DE: dichloromethanolic extracts; EE: ethanolic extracts; R: roots; F: flowers; F1: phenol/carboxylic acid fraction; F2: alkylamide fraction. Scale bar = 50 µm.

References

1. Cech, N.B.; Eleazer, M.S.; Shoffner, L.T.; Crosswhite, M.R.; Davis, A.C.; Mortenson, A.M. High performance liquid chromatography/electrospray ionization mass spectrometry for simultaneous analysis of alkamides and caffeic acid

derivatives from Echinacea purpurea extracts. *J. Chromatogr. A* **2006**, *1103*, 219–228, doi:https://doi.org/10.1016/j.chroma.2005.11.008.

2. Spelman, K.; Wetschler, M.H.; Cech, N.B. Comparison of alkylamide yield in ethanolic extracts prepared from fresh versus dry Echinacea purpurea utilizing HPLC–ESI-MS. *J. Pharm. Biomed. Anal.* **2009**, *49*, 1141–1149, doi:https://doi.org/10.1016/j.jpba.2009.02.011.
3. Pellati, F.; Epifano, F.; Contaldo, N.; Orlandini, G.; Cavicchi, L.; Genovese, S.; Bertelli, D.; Benvenuti, S.; Curini, M.; Bertaccini, A.; et al. Chromatographic methods for metabolite profiling of virus- and phytoplasma-infected plants of Echinacea purpurea. *J. Agric. Food Chem.* **2011**, *59*, 10425–10434, doi:10.1021/jf2025677.
4. Thomsen, M.O.; Fretté, X.C.; Christensen, K.B.; Christensen, L.P.; Grevsen, K. Seasonal variations in the concentrations of lipophilic compounds and phenolic acids in the roots of Echinacea purpurea and Echinacea pallida. *J. Agric. Food Chem.* **2012**, *60*, 12131–12141, doi:10.1021/jf303292t.
5. He, X.; Lin, L.; Bernart, M.W.; Lian, L. Analysis of alkamides in roots and achenes of Echinacea purpurea by liquid chromatography–electrospray mass spectrometry. *J. Chromatogr. A* **1998**, *815*, 205–211, doi:https://doi.org/10.1016/S0021-9673(98)00447-6.
6. Mudge, E.; Lopes-Lutz, D.; Brown, P.; Schieber, A. Analysis of alkylamides in Echinacea plant materials and dietary supplements by ultrafast liquid chromatography with diode array and mass spectrometric detection. *J. Agric. Food Chem.* **2011**, *59*, 8086–8094, doi:10.1021/jf201158k.
7. Bauer, R.; Remiger, P. TLC and HPLC analysis of alkamides in Echinacea drugs. *Planta Med.* **1989**, *55*, 367–371, doi:10.1055/s-2006-962030.
